# The effect of using fins in different configurations on the performance of adsorption refrigeration system

**DOI:** 10.1038/s41598-025-17060-8

**Published:** 2025-09-12

**Authors:** Samah I. Hatab

**Affiliations:** Department of Mechanical Engineering, Faculty of Engineering, Suez University, P. O. Box: 43221, Suez, Egypt

**Keywords:** Different fins configurations, Adsorption refrigeration system, Finned generator, Adsorption cycle enhancement., Mechanical engineering, Solar thermal energy

## Abstract

In this experimental investigation, the impact of six different configurations of applied fins in an adsorption refrigeration system utilizing an AC/Ethanol pair was examined. Fins in six different configurations (straight and inclined fins of one sector, along with fins of two, three, four, and six sectors) are utilized to improve heat and mass transfer within the generator. The experiments are carried out under non-equilibrium conditions, with the evaporating, generating, and condensing temperatures established at 5 °C, 82 °C, and 22 °C, respectively. The findings revealed that the inclined fins of one sector configuration achieved the highest coefficient of performance (COP) at 0.44, representing a 25.7% improvement over pure activated carbon (AC) without fins. Additionally, the fins configured in a three-sector arrangement exhibited the greatest specific cooling effect (SCE), uptake amount (Δx), and reduction in evaporating temperature, measuring 44.5 kJ/kg, 0.06 kg/kg, and 19.5 °C, respectively, with enhancement rates of 71.2%, 87.5%, and 95%, respectively, when compared to pure activated carbon (AC) without fins. A significant finding of this study is the variation in the impact of each fins configuration on the transfer of heat and mass within the generator, as well as the performance of the adsorption system.

## Introduction

A multitude of studies have employed solar energy as a sustainable resource to improve the efficiency of adsorption refrigeration systems. Baiju & Muraleedharan^1^ introduced a solar adsorption refrigeration system that utilizes activated carbon-methanol as the working pair. The experimental findings indicated that the system achieved a coefficient of performance (COP) of 0.196 during the day and 0.335 at night. Additionally, the specific cooling power (SCP) values recorded were 47.83 W/s.kg during the daytime and 68.2 W/s.kg at nighttime.

Rekiyat Suleiman et al.^2^ demonstrated the modeling and simulation of a solar adsorption refrigeration system that utilizes a flat plate solar collector along with activated carbon-methanol as the working pair. A transient simulation of the cooling system was executed with an evaporator temperature set at 0 °C and a condenser temperature at 25 °C, employing the TRNSYS 16 software over the course of a typical year. The findings of the study indicated a refrigeration effect value of 4814.83 kJ, a solar coefficient of performance (COPs) of 0.024, a cooling coefficient of performance (COP) of 0.608, and a heating efficiency of 0.46. The system successfully achieved a cold room temperature of approximately 1 °C.

Ankush Kumar Jaiswal et al.^3^ introduced a dynamic analysis of a single-stage, two-bed silica gel + water adsorption chiller, utilizing an evacuated tube solar collector field with a hot water temperature of approximately 95 ˚C. This analysis was conducted during the months of April (summer) and December (winter). The research concentrated on how variations in the collector area and the adsorption cycle duration influenced the system’s performance. The findings indicated that by appropriately selecting the collector area and cycle duration, both the solar coefficient of performance and the cooling capacity could be optimized.

Mohand Berdja et al.^4^ developed a prototype of an adsorption tube collector designed for cooling and solar energy that is directly absorbed, employing activated carbon-methanol as the working pair. The thermal coefficient of performance (COP) was determined to be around 0.49, while the solar COP was measured at 0.081.

Fardousi Ara Begum et al.^5^ introduced a two-bed adsorption chiller powered by solar heat with an intermittent heat source, utilizing silica gel-water as the working pair. The findings indicated that a cycle time of 1100 s, combined with a collector area of 38.64 m^2^, resulted in enhanced performance while maintaining a constant chilled water outlet temperature of 7 °C. Ambarita and Kawai^6^ evaluated the efficacy of the solar adsorption refrigeration system employing various adsorbents in conjunction with methanol and a flat-plate solar collector measuring 0.5 m × 0.5 m. The generator was filled with 100% activated alumina (referred to as 100AA), a mixture of 75% activated alumina and 25% activated carbon (75AA), a mixture of 25% activated alumina and 75% activated carbon (25AA), and 100% activated carbon. The results revealed that the coefficients of performance (COP) for 100AA, 75AA, 25AA, and 100AC were 0.054, 0.056, 0.06, and 0.074, respectively. Furthermore, the use of activated carbon demonstrated superior performance compared to activated alumina.

Umair et al.^7^ conducted a simulation study on a solar adsorption refrigeration system utilizing a wing-type compound parabolic concentrator. The objective of the study was to assess the behavior of the system and to compare the performance between the wing-type and linear systems. The simulation findings indicated that the performance of the system with a wing-type CPC improved by as much as 6% during the summer and 2% in the winter, in comparison to the performance of a linear CPC with an equivalent collector length. Additionally, ice production was found to increase by up to 13% in the summer when using the wing-type CPC. Dhokane Nilesh et al.^8^ developed an alternative eco-friendly adsorption refrigeration cycle designed to achieve temperatures typically found in conventional refrigerators, while being independent of electric power and incurring zero operational costs. Yuan et al.^9^ introduced an adsorption refrigeration system that incorporates a concentrated solar collector. This study employed two types of zeolite with water as the working pairs. The coefficient of performance (COP) and specific cooling power (SCP) of the SAPO-34 zeolite surpassed those of the ZSM-5 zeolite, achieving a maximum COP of 0.169. A study conducted by Li et al.^10^ utilized simulation to analyze the heat transfer characteristics of an evacuated tube in a solar-powered adsorption refrigerator. The aim of this research was to identify the optimal diameter of the tubes and the spacing between two adjacent tubes.

Jingkang Liang et al.^11^ identified enhanced mass transfer as a primary approach to augment the efficiency of a solar adsorption chiller, which incorporates two condensers and a micro-vacuum pump to further reduce the desorption pressure. The research employed activated carbon-methanol as the working pair, testing three distinct cooling temperatures of 20 °C, 30 °C, and 40 °C, respectively. The findings revealed that lowering the cooling temperature significantly enhances the coefficient of performance (COP) in the context of enhanced mass transfer, with the system achieving a COP of 0.169. Erek et al.^12^ conducted a detailed analysis of how variations in fin geometry affect heat transfer and pressure drop within the system, utilizing a computational fluid dynamics (CFD) program. Their results demonstrate that placing the fin tube downstream markedly improves heat transfer efficiency, and that increasing the ellipticity of the fin tube further enhances heat transfer.

Moreover, Louajari et al.^13^ and AL Mers et al.^14^ created a model for a cylindrical finned reactor intended for an adsorption cycle that utilizes a combination of AC and ammonia. Louajari et al. concentrated on the sensitivity of the coefficients of performance (COPs) concerning the geometric parameters of the reactor. Furthermore, AL Mers et al. demonstrated that the optimal diameter, the mass cycled, the maximum temperature achieved in the finned adsorber, and the efficiency of the solar adsorption refrigeration system with a fin-equipped adsorber are superior to those of the system without fins. Ilis et al.^15^ proposed an adsorption process that utilized a numerical solution to evaluate the cycle’s performance. The results obtained indicated that the heat transfer rate within an adsorbent bed can be improved by the presence of fins.

Rezk and AL-Dadah^16^ developed a simulation model for the chiller to investigate how variations in fin spacing affect chiller performance. Their findings indicated that the minimum permissible spacing enhanced cooling capacity by 3%, although this was accompanied by a 2.3% reduction in the coefficient of performance (COP). Additionally, they identified that an increased cycle time could boost the chiller’s cooling capacity by 8.3%, which was used to ascertain the optimal cycle time for achieving maximum cooling capacity. Ameel et al.^17^ examined a proposed correlation related to complex interrupted fin designs, including louvered fins, which led to an overestimation of fin efficiency through a novel methodology. Ji et al.^18^ created a finned-tube adsorbed bed using a large-diameter aluminum alloy, utilizing a working pair of AC and methanol. Their experimental outcomes demonstrated an ice production rate of 6.5 kg per day, along with a coefficient of performance (COP) of 0.122. Furthermore, the cooling efficiency of the solar-powered adsorption refrigeration system featuring valve control during the adsorption/desorption process was markedly superior to that of the system lacking valve control.

In a distinct investigation, Ameel et al.^19^ examined a range of fin geometries and heat exchanger configurations of varying lengths to develop a comparative framework for heat exchangers with an arbitrary number of tube rows, while also considering the impact of longitudinal tube pitch. Atiya et al.^20^ performed a study on a finned-tube adsorption bed heat exchanger designed for air conditioning applications using silica gel. This research, which operated on a cycle duration of 40 min, involved hot and cooling water temperatures of 90 °C and 30 °C, respectively, along with an evaporating temperature of 11.4 °C. The results revealed a specific cooling power (SCP) of 163 W/kg and a coefficient of performance (COP) of 0.16.

In a related investigation, Ramji et al.^21^ examined the influence of wall thickness on the cooling efficiency and desorption temperature within an air conditioning system. This study utilized computational fluid dynamics (CFD) to model an AC/methanol working pair situated in an adsorption bed heat exchanger, which was defined by a bed temperature of 120 °C and a wall thickness of 2 mm. The simulation, which operated over a cycle duration of 1200 s, produced a cooling power of 0.65 kW and a coefficient of performance (COP) of approximately 0.25. Additionally, a model for an adsorption cooling system employing an AC/methanol combination was proposed by EL Fadar^22^ and corroborated with experimental data. The findings indicated that increasing the number of fins from 15 to 20 not only enhanced heat and mass transfer but also improved the coefficient of performance (COP), establishing an optimal fin spacing range between 4 and 5.2 cm.

Mohammed et al.^23^ further improved system efficiency by utilizing a composite adsorbent. Silica-gel particles were incorporated into a high-porosity aluminum foam, which was then arranged in a silica gel-water-packed bed. The findings indicated that aluminum foam with 20 PPI (pores per inch) is optimal for adsorption cooling applications due to its elevated surface area and reduced cell size. This 20 PPI aluminum foam can achieve an SCP of 827 W/kg, a CP v of 517 W/m 3, and a COP of 0.75. Cheppudira et al.^24^ implemented an adsorption refrigeration system featuring three enhanced tubes with varying fin geometries (including fin spacing and fin height). The results demonstrated that the enhanced tubes exhibit external convective heat transfer resistances that are 9.8–21 times lower compared to the plain tube. Furthermore, the enhanced tube with the greatest fin height shows a 33% reduction in external convective resistance compared to those with shorter fin heights, thereby improving the overall specific cooling power (SCP).

Li et al.^25^ investigated a finned adsorption bed utilizing the SAPO-34 zeolite-water combination as the working pair, varying the number of fins from 2 to 8 while maintaining a constant fin height and thickness. The findings indicated that the insertion of fins significantly enhanced heat transfer and increased solar energy input during both the preheating and desorption phases for the cases with enhanced fins. Overall, the cycle performance results were found to be advantageous. In a separate study conducted by Zhao et al.^26^, activated carbon-methanol was employed as the working pair within a solar adsorption refrigeration system, which was simulated using a novel adsorbent bed featuring finned tubes. The study concluded that the presence of fins substantially influenced the thermal performance of the adsorption system. Khatibi et al.^27^ examined the impact of incorporating aluminum additive particles into an unconsolidated adsorbent bed on the efficiency of an adsorption cooling system utilizing a finned flat-tube heat exchanger filled with silica gel SWS-1 L. The results indicated that beds with fin heights of 8 mm and 20 mm, filled with optimal particle diameters, and additive volume fractions of 15% and 20%, respectively, maximized the volumetric cooling power (VCP) to 370.8 and 399.7 kW/m³ based on the total volume of the adsorber heat exchanger.

Golparvar et al.^28^ conducted a study utilizing a three-dimensional non-equilibrium model to examine the impacts of heat and mass transfer within annular and longitudinal finned tube adsorber beds that are filled with zeolite-13x particles. The research focused on how fin height and spacing influence the operational parameters of the system. The findings indicate that an annular finned tube ABHEx delivers a total cooling power that is 10% greater than that of a longitudinal finned tube ABHEx when the fin spacing is optimized. Mahmoud et al.^29^ carried out an investigation into the efficacy of a small-scale two-bed adsorption chiller that is integrated with a heat and mass recovery cycle. This model was employed to evaluate the influence of fin design parameters on heat transfer within the bed and the overall performance of the system. The working pair utilized in the simulations was RD silica gel–water. The results indicated that reducing the fin spacing significantly enhances heat transfer, with the specific cooling capacity improving by an average of up to 147.6% when the fin spacing is decreased from 4 mm to 2 mm. Conversely, increasing the fin radius results in lower temperatures of the adsorbent. Additionally, the specific cooling capacity improved by an average of up to 44% when the fin radius was reduced from 16 mm to 8 mm. It was also noted that the fin thickness has a negligible effect on heat transfer within the bed.

Shanshan et al.^30^ utilized three varieties of conventional finned tube adsorption beds to enhance the heat and mass transfer in adsorption refrigeration systems. The findings demonstrated that the overall efficiency of the system employing a finned tube adsorption bed with an annular outer fin surpasses that of a plate outer fin and an annular inner fin. Furthermore, the perforated fin design significantly improved the refrigeration performance of the finned tube adsorption bed. Palash et al.^31^ conducted a study focused on the development of an annular finned tube adsorber model that functions as a thermal compressor within adsorption refrigeration systems. The generalized model was simulated using an activated carbon–methanol working pair. The system achieved a maximum Coefficient of Performance (COP) of 0.3706 at a regeneration temperature of 353 K and an evaporator temperature of 283 K. Additionally, the highest Specific Cooling Power (SCP) recorded was 144.8 W/kg at an evaporator temperature of 283 K and a regeneration temperature of 393 K. Ibrahim et al.^32^ performed a numerical investigation into the performance of a wire-finned tube adsorber bed integrated with an aluminum fumarate metal-organic framework material, utilizing either coating, packing, or a combination of both methods. The results indicated that the coated wire-finned heat exchanger outperformed both the packed and the combined packed and coated configurations in terms of specific daily water production (SDWP) and specific cooling power (SCP). It was also found that employing a tube diameter of 22 mm, a fin height of 20 mm, 44 fins per loop, and a coating thickness of 0.5 mm could yield a maximum daily water production of 300 L/day and a cooling power of 8.3 kW.

Mahmoud et al.^33^ conducted a numerical investigation into the application of an innovative aluminum foamed bed filled with advanced Maxsorb adsorbent within a two-bed adsorption system. Their findings indicate performance enhancements when compared to the traditional finned-tube-based system utilized for ice production. The Al-foam-based system exhibited superior performance at a foam thickness of 2 mm, achieving a maximum ice production of 49 kg_ice_/kg_ads_ in 8 h, which represents a 26.6% improvement over the finned-tube-based counterpart at a cycle time of 400 s. The optimal coefficient of performance (COP) of 0.366 was recorded at a foam thickness of 5 mm and a cycle time of 1200 s, reflecting a 26.7% increase. However, the effective uptake of the Al-foam-based system significantly declined at a foam thickness of 10 mm, leading to a deterioration in the system’s overall performance. Pooriya et al.^34^ introduced a design for an adsorption/desorption (A/D) heat exchanger (HE) featuring longitudinal fins within the sorption bed to improve heat transfer during methanol adsorption/desorption processes in activated carbon (AC). The finned HE design resulted in a 1.8 °C rise in the maximum bed surface temperature and a reduction in process time by 3 h and 40 min when compared to the non-finned HE. Further experiments with the finned HE at varying water flow rates (5, 10, and 15 L/min) revealed enhanced heat transfer at a flow rate of 15 L/min, attributed to the promotion of turbulent flow. Helmy et al.^35^ performed an experimental study aimed at enhancing thermal performance by increasing the thermal conductivity of the adsorption bed. This was achieved by incorporating 10%, 20%, and 30% metallic copper powder with activated carbon to augment the bed’s thermal conductivity. The results indicated that a copper filling with a mass concentration of 20% was the optimal metallic additive ratio for improving the system’s thermal performance. This modification lowered the evaporator temperature to −5 °C and − 10 °C for heating water flow rates of 3 L/min and 2 L/min, respectively. Additionally, it improved the cycle COP of the system by 49% and 46% at hot water flow rates of 2 L/min and 3 L/min, respectively. The highest cycle COP achieved by the current system was 0.92 under the condition of 20% additives at a hot water flow rate of 3 L/min.

Previous studies have shown that some investigations focused on the effectiveness of adsorption systems that utilize solar collectors, while another research effort examined how the geometrical parameters of fins (including their number, height, and spacing) affect the performance of the adsorption system. These investigations have played a significant role in validating the impact of fins on enhancing heat transfer, identifying specific characteristics, and clarifying the acceptable limit values observed in their results for COP, SCE, and SPC. It is important to highlight that the previous studies did not explore the particular research focus of this study, which involves the application of fins with different configurations concerning sectors and angles.

The main aim of this research is to experimentally assess the performance of a transient intermittent finned adsorption refrigeration system by implementing six different fin configurations. Furthermore, the finned generator is designed to improve heat and mass transfer within the solid layers, thus enhancing the overall efficiency of the system. The most effective configuration was identified and compared against the system’s performance using pure AC without fins, facilitating an evaluation of the improvement rate. The six fin configurations analyzed included fins with one sector (both straight and inclined) as well as two, three, four, and six sectors. This study seeks to identify the optimal fin configuration that should be utilized to achieve the highest performance.

## Materials and methods

The adsorption refrigeration system was designed, built, and integrated to reduce energy losses linked to heating and cooling processes that occur during the pressure cycle transition from the evaporator to the condenser and vice versa. This system employs three valves positioned in front of the generator, evaporator, and condenser components. The design of this system facilitates accurate control and assessment of the varying conditions related to adsorption and desorption within the generator.

### The experimental setup

The adsorption refrigeration system comprises three primary components: the generator, condenser, and evaporator, along with auxiliary components such as water tanks, vacuum and hot water pumps, stainless steel valves, ball valves, stainless steel connecting tubes, heaters, and connecting hoses. Additionally, it includes measuring devices like liquid indicators, pressure gauges, and temperature thermocouples.

**Fig. 1:**
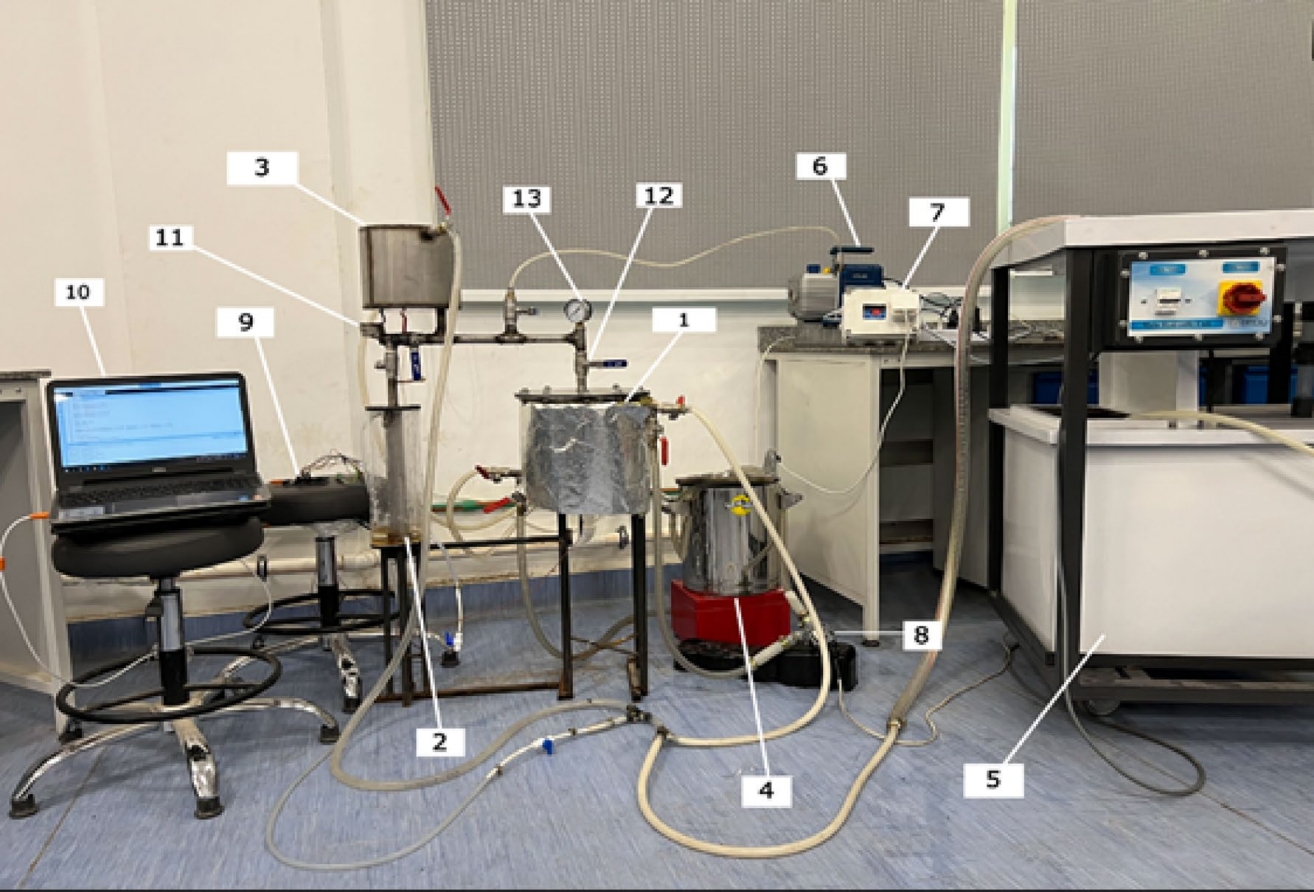
Pictorial View of adsorption refrigeration system. **1**. Generator, **2.** Evaporator, **3.** Condenser, **4.** Hot Water Tank,**5.** Cold Water Tank, **6.** Vacuum Pump, **7.** Control Unit, **8.** Hot water Pump, **9.** Arduino Mega Board, **10.** Data Acquisition, **11.** Capillary Tube, **12.** Ball Valve, **13.** Pressure Gauge.

**Fig. 2:**
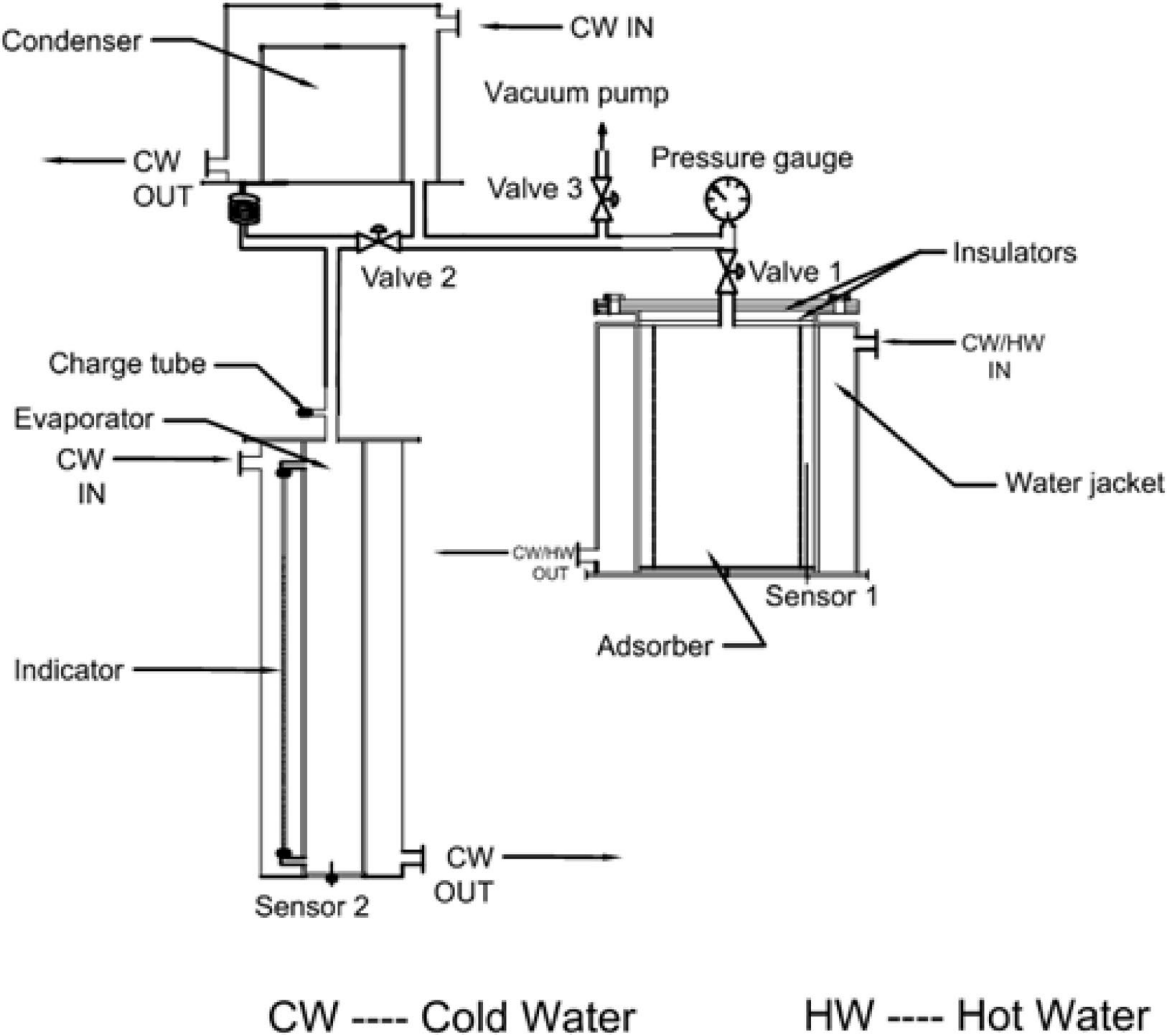
Schematic drawing of adsorption refrigeration system.

The refrigeration cycle, utilizing 0.5 kg of absorbent, is specifically designed to function with a dedicated evaporator, as illustrated in Figs. 1 and 2. This evaporator consists of an inner stainless steel cylinder and is controlled by two stainless steel valves. Stainless steel tubes link the evaporator to both the generator and condenser, creating a compact system. To assess the quantity of ethanol that is adsorbed or desorbed during the adsorption-desorption processes, a graduated glass tube is affixed to the evaporator, serving as a liquid level indicator.

**Fig. 3:**
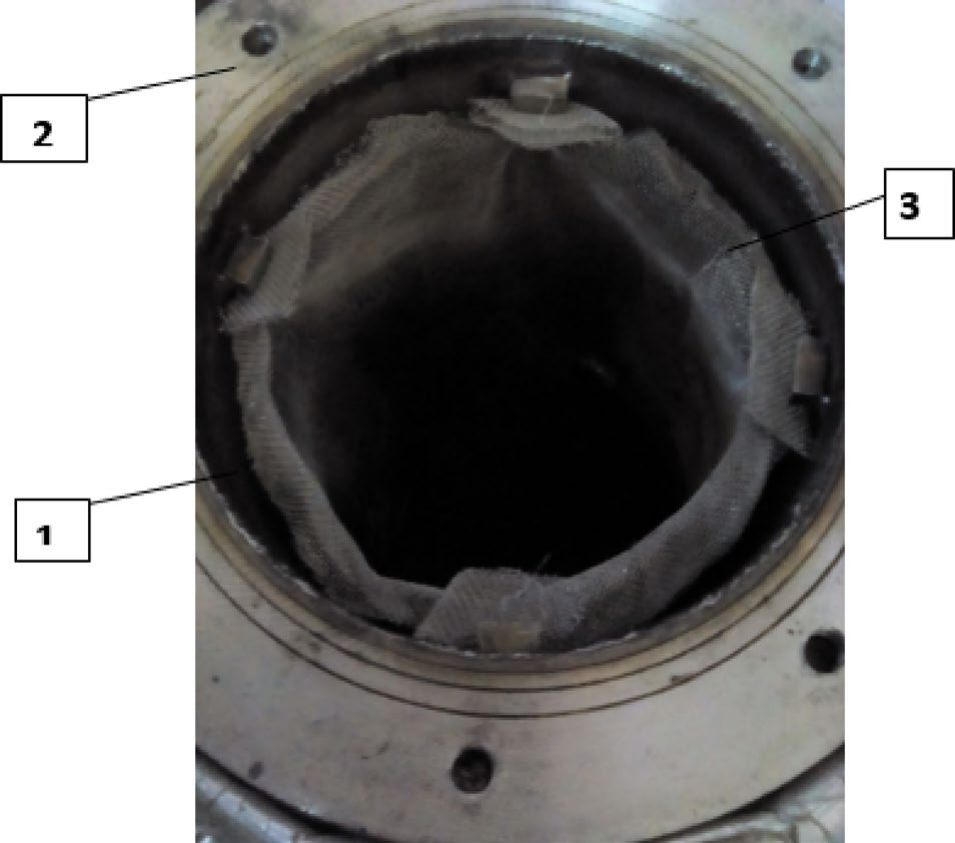
Illustrated interior view of generator filled with activated carbon. **1**. Adsorbent (AC) column, **2**. Generator, **3**. The mesh.

**Fig. 4:**
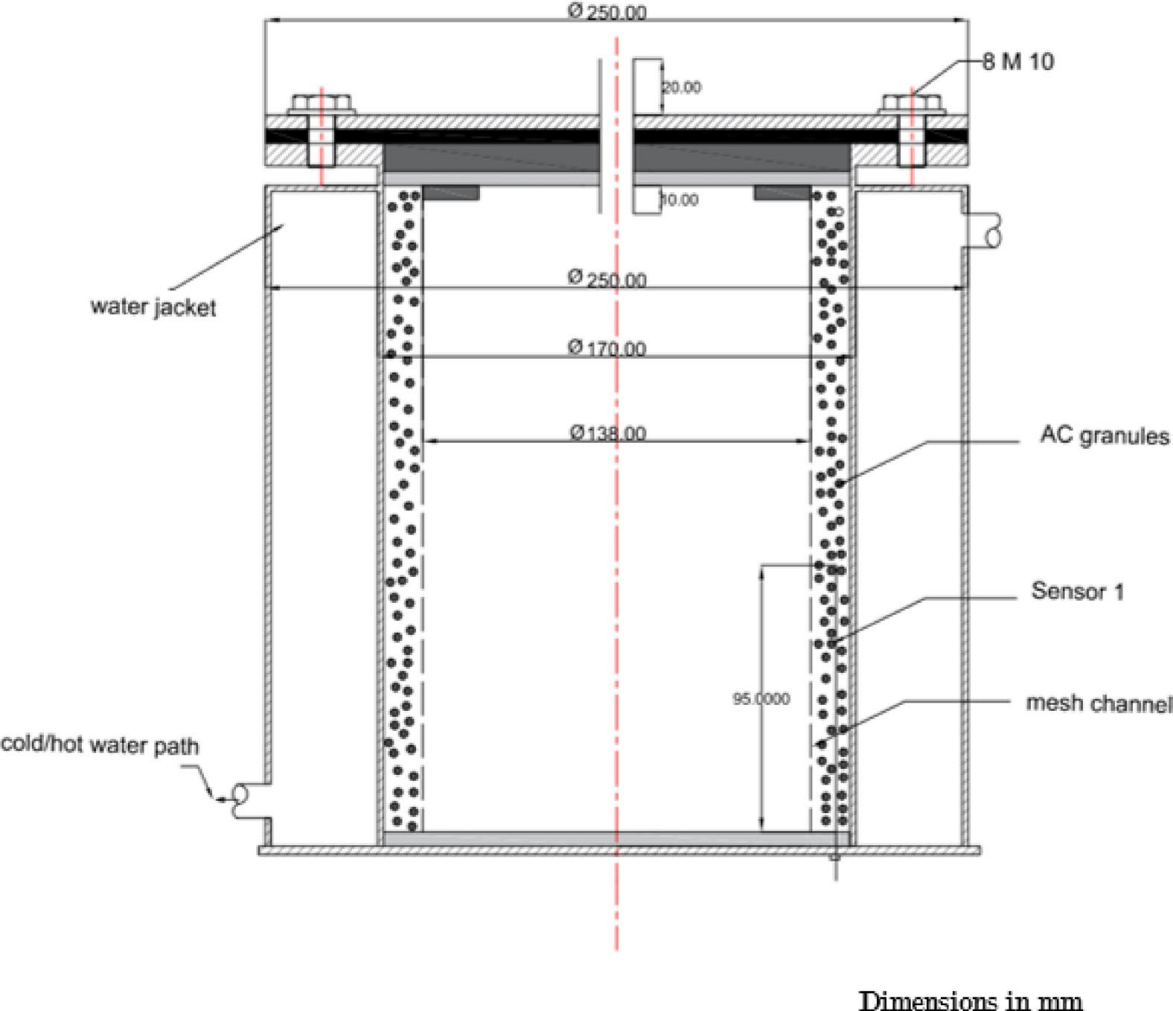
Cross-section of generator showing the position of AC granules.

The finned generator is composed of three primary stainless steel cylinders: the water jacket, the adsorber, and the porous cylinder, with diameters measuring 250 mm, 170 mm, and 138 mm, respectively. The space between the adsorber cylinder and the mesh is filled with AC granules, creating an adsorbent column that is 220 mm height and 16 mm thick, as illustrated in Figs. 3 and 4. This adsorbent column consists of layers of AC granules, with the outer layer making contact with the inner surface of the adsorber cylinder. This interaction facilitates the transfer of heat or cold to the granules via the hot or cold water circulating within the water jacket cylinder.

**Fig. 5:**
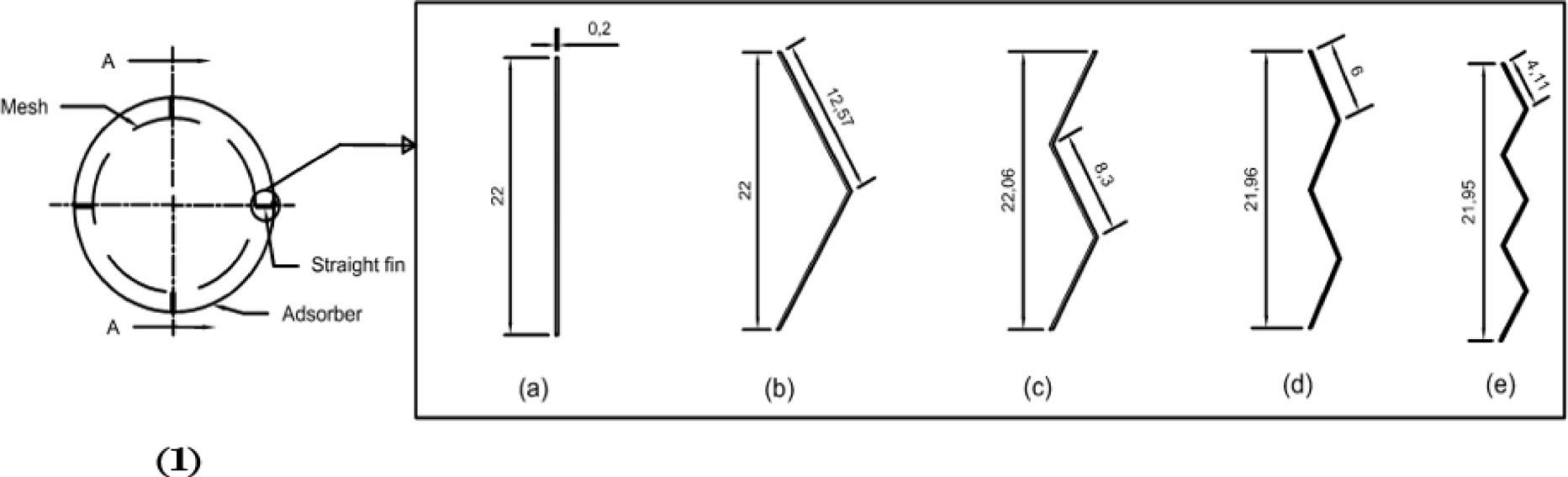
Schematic drawing of the generator, (**1**) Plan View, and Section Side View at A-A of fins in different configurations, (**a**) one sector, (**b**) two sectors, (**c**) three sectors, (**d**) four sectors, (**e**) six sectors.

**Fig. 6:**
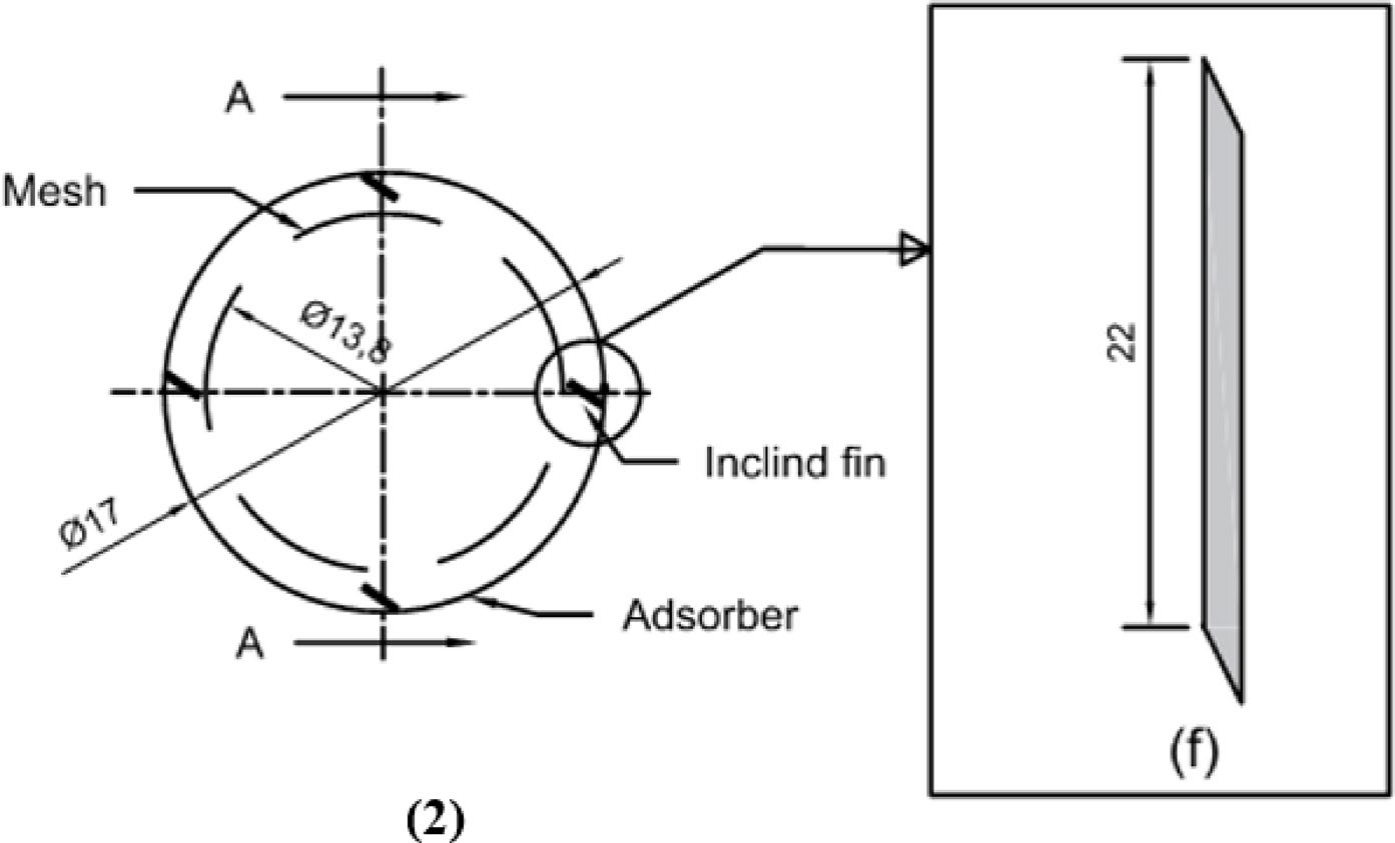
Schematic drawing of the generator, (**2**) Plan View, and (**f**) Section Side View of inclined aluminum fin at A-A.

A stainless steel mesh is utilized to hold the adsorbent sample (AC) in place while allowing the refrigerant to pass through during the adsorption and desorption phases. Furthermore, four aluminum fins are placed at equal intervals within the adsorbent column throughout the generator cylinder. The fins examined in this research are configured in six distinct arrangements (straight in one sector, inclined in one sector, two sectors, three sectors, four sectors, and six sectors), as depicted in Figs. 5 and 6. Each fin configuration has dimensions of 22 mm in height, 16 mm in width, and 2 mm in thickness. The fins configured in two, three, four, and six sectors possess a vertical length of 20 cm within the generator, while their lengths prior to the formation of the sectors are approximately 25 cm.

**Fig. 7:**
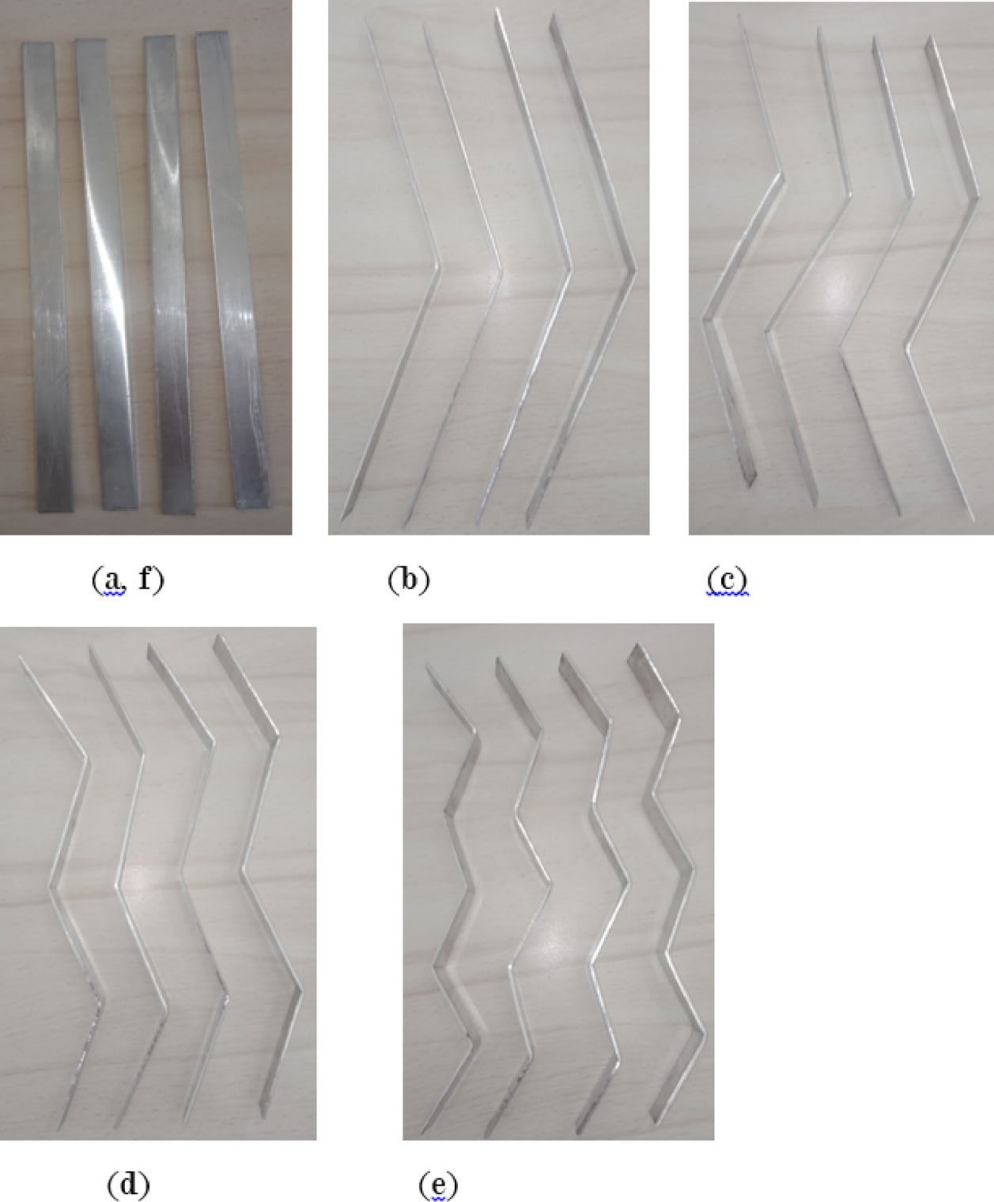
Photos of fins in different configurations.

In Fig. 7, each fin configuration consists of sectors that are of equal lengths. Furthermore, the present study illustrated the impact of the angle of fin positioning on system performance, as evidenced by the scenarios involving both straight and inclined fins within one sector. These configurations have been evaluated as elaborated upon in the results section.

The four fins are positioned in a radial arrangement and are held in place using AC granules. Additionally, they are anchored along the length of the generator with a metal stopper that is welded 22 cm from the bottom of the generator. The fin is situated between the upper stopper and the base of the generator to guarantee optimal contact with the inner surface of the absorber cylinder, as illustrated in Figs. 5 and 6. Research conducted by Zepeng Wang et al.^36^ determined the ideal number of fins for installation within the generator, advocating for the use of four fins to achieve the highest level of enhancement. For the ethanol gas liquefaction during the desorption process, the condenser was designed as a heat exchanger. It features a stainless steel (SS) valve at the inlet that opens at the onset of the desorption process. The condenser is comprised of two SS cylinders, with the inner cylinder serving to condense vapor refrigerant and the outer cylinder facilitating the circulation of cold water within. The stainless steel water jacket cylinders encasing the generator and condenser are utilized for both cold and hot water flows, effectively cooling and heating these components throughout the adsorption and desorption processes. For laboratory experiments, hot water was produced using a 1 kW heater, with the heating source serving as a simulation of solar energy.

For the purpose of cycle measurements, the cycle pressure is monitored using a pressure gauge placed above the generator, which is the nearest location to the generator itself. This gauge has an accuracy of ± 0.4 and operates within a range of 0 to 100 kPa. In terms of temperature measurements, T-type thermocouples were employed, exhibiting a precision of 0.5. These sensors were installed within the generator and evaporator, specifically from the bottom surface (sensors 1 and 2), as illustrated in Figs. 2 and 4. Sensor 1 is positioned at half the height of the generator to ensure accurate readings. Conversely, sensor 2 is located in the evaporator at a height of only 1 cm to provide precise measurements of any liquid refrigerant present.

The assessment of the quantity of ethanol that is adsorbed or desorbed was conducted using a graduated glass liquid level indicator, which was calibrated with a known volume of liquid utilizing a syringe with a precision of 0.5 ml. This liquid indicator, constructed from transparent glass, has a thickness of 2 mm, a diameter of 3 mm, and a height of 300 mm. Within the compact cycle, the measuring instruments for temperature, pressure, and ethanol content were arranged as depicted in Figs. 1 and 2.

### Experimental procedure

**Fig. 8:**
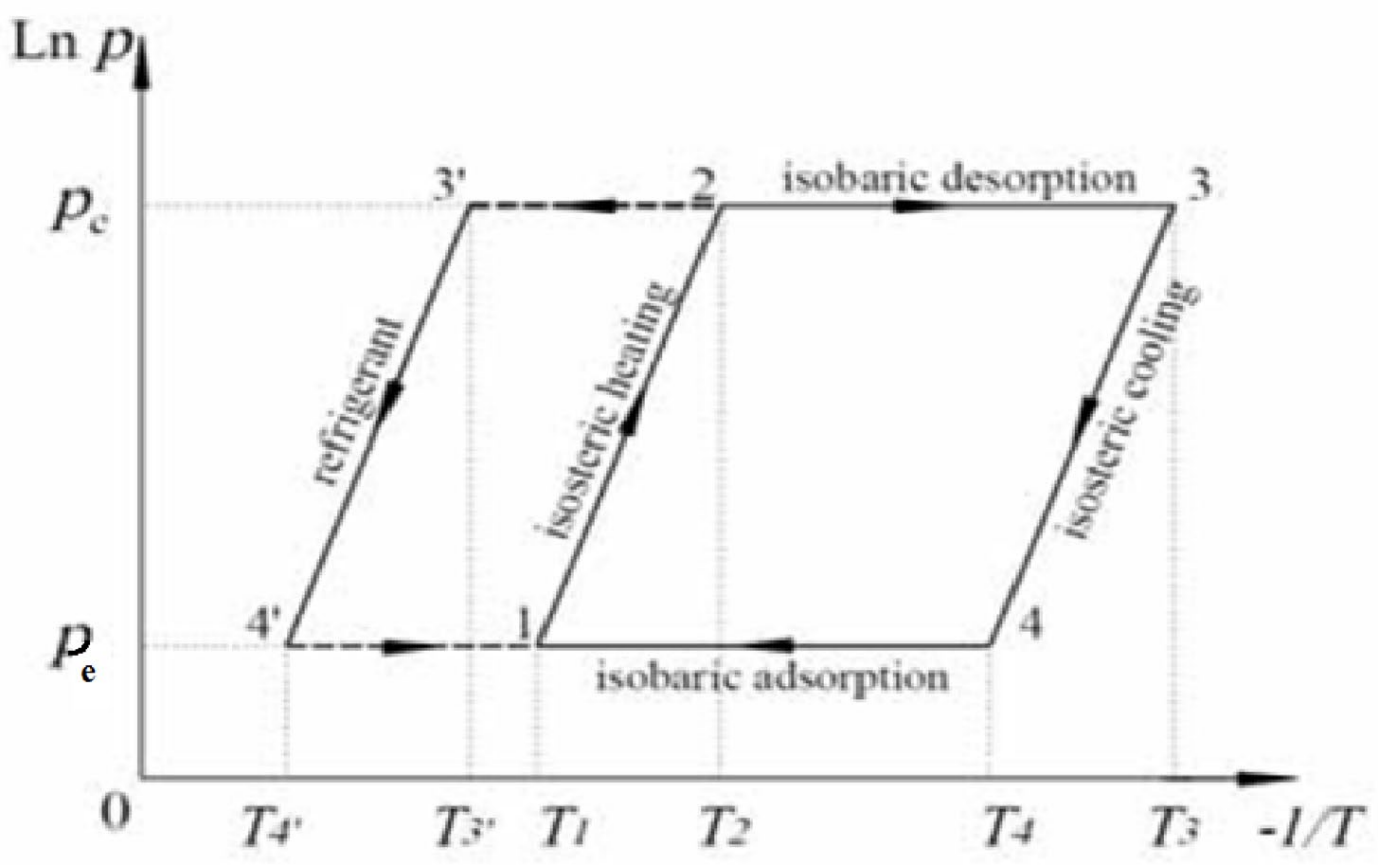
Clapeyron diagram of an ideal adsorption refrigeration cycle.

An adsorption refrigeration system was utilized for the experimental procedures. The adsorption cycle underwent evacuation and degassing for a duration of 24 h to confirm the absence of moisture or leakage. Following this, the generator was filled with 0.5 kg of activated carbon (AC) and heated using water circulated through the generator’s water jacket. The generator was then subjected to vacuum degassing for an additional 12 h to ensure it remained moisture-free. Subsequently, the generator was calibrated to a condensing pressure of 6 kPa by heating the AC column to 82 °C at point 3, as illustrated in Fig. 8. In the subsequent step, a suitable amount of ethanol was introduced into the evaporator, considering the refrigerant volume evacuated from the evaporator during the evacuation process to achieve an evaporator pressure of 2 kPa. The cooling process commenced by cooling the generator until the evaporating pressure was reached at point 4. At this juncture, the adsorption process initiated, and valve 2 was opened to connect the evaporator with the generator, facilitating the movement and adsorption of liquid ethanol by the AC within the generator. Upon completion of the adsorption process at point 1, the generator was heated to the condensing pressure to commence the desorption process at point 2. The condensed ethanol began to be recaptured in the evaporator until the desorption process ceased at the maximum cycle temperature at point 3. Throughout the adsorption and desorption processes, all readings of pressure, temperature, and ethanol quantity were meticulously recorded.

### Thermo physical properties of working pair and aluminum fins used

Throughout the experiments aimed at investigating the thermo-physical properties of the working pair, activated carbon was utilized as the adsorbent, as illustrated in Table 1. The activated carbon samples demonstrated a relatively low total pore volume and thermal conductivity, along with a restricted BET surface area, which considerably affected their efficacy during the adsorption-desorption cycle. Ethanol, noted for its distinct thermal properties, was employed as the refrigerant in all conducted experiments. The thermo-physical attributes of the working pair were obtained from the manufacturer, as shown in Table 1. In this study, six distinct fin configurations were utilized. The physical characteristics of each configuration are detailed in Table 2. Additionally, their positioning and orientation angles are represented in Figs. 5 and 6. The generator is fitted with four fins, which are constructed from aluminum, a material recognized for its superior thermal conductivity, approximately 237 W/m K, thus improving heat transfer both within the generator and through its water jacket.

The present study seeks to explore various fin configurations to enhance heat and mass transfer among activated carbon layers within the generator. The objective is to identify the optimal configuration that maximizes heat and mass transfer, thereby achieving the lowest cooling temperature of the cycle while minimizing energy consumption and maximizing performance within the shortest cycle duration. Fins with diverse configurations were employed, including a 45-degree orientation for inclined fins in a single sector. These designs exhibit varying responses to heat and mass propagation within the generator. Consequently, minor adjustments to the fin design can lead to enhancements without incurring additional costs during construction or operational cycles. This results in improved performance coefficients, which are influenced by cooling temperature and cycle duration, all while maintaining minimal energy consumption. Most fin configurations demonstrate enhanced cycle performance compared to pure activated carbon without fins, although their efficiencies vary as elaborated in the results section.

 The experimental operating conditions utilized in this study are detailed in Table 3. The maximum generating temperature within the cycle is 82 °C, corresponding to a condenser pressure of 6 kpa. Conversely, the minimum evaporator temperature required in the cycle is 5 °C, which is associated with an evaporating pressure of 2 kpa. These parameters are implemented on an adsorbent column with a thickness of 16 mm. This reduced thickness facilitates a shorter cycle duration.

**Table 1:** Thermo-physical properties of granular activated carbon (AC) and ethanol.

Properties	Value
Bet surface area of AC, m^2^/g	7 ˟ 10^2^
Total pore volume of AC, cc/g	0.35
Average pore diameter of AC, nm	2.19
Thermal conductivity of AC, W/m K	1.6
Apparent density of AC, kg/m^3^	450
Specific heat of AC, kJ/kg K	0.9
Ethanol boiling point, ^0^C	78.3
Ethanol molecular weight	46.07
Ethanol purity, %	99.9
Specific heat of ethanol, kJ/kg K	2.44
the latent heat of vaporization of ethanol, kJ/kg	842

**Table 2:** Physical characteristics of fins in six configurations.

Case No.	Fins configuration	Total Height, (mm)	Width, (mm)	Thickness, (mm)	Sector length, (mm)
1	Fins of one sector, (straight fins)	220	16	2	220
2	Fins of one sector, (inclined fins)	220	16	2	220
3	Fins of two sectors	220	16	2	12.57
4	Fins of three sectors	220.6	16	2	8.3
5	Fins of four sectors	219.6	16	2	6
6	Fins of six sectors	219.5	16	2	4.11

**Table 3:** The operating parameters of the experiments.

Parameter	Value
Required evaporating temperature, T_e_ (^o^C)	5
Cooling water temperature, T_c_ (^o^C)	22
Generating temperature, T_g_ (^o^C)	82
Ambient temperature, T_am_ (^o^C)	22
Corresponding evaporating pressure, P_e_ (kPa)	2
Corresponding condensing pressure, P_c_ (kPa)	6

### Performance parameters

The coefficient of performance (COP) and specific cooling effect (SCE) are recognized as superior indicators for assessing the efficacy of the adsorption system, as highlighted by Sumathy and Zhongfu^37^. The performance of the adsorption refrigeration system is defined by the coefficient of performance, as demonstrated in Eq. (1).

1 $$COP = \frac{{Q_{r} }}{{\left( {Q_{{1 - 2}} ~ + ~Q_{{2 - 3}} } \right)}} = ~\frac{{\Delta M_{r} ~L_{o} ~{-}~c_{r} ~\Delta M_{r} ~\left( {T_{3^\prime} - T_{4^\prime} } \right)}}{{~\left[ {\left( {c_{{ac}} ~M_{{ac}} ~ + ~c_{r} ~M_{{r1}} } \right)\left( {T_{2} - T_{1} } \right)} \right] + \left[ {\left( {c_{{ac}} ~M_{{ac}} ~ + ~c_{r} ~M_{{r~av}} } \right)~\left( {T_{3} - T_{2} } \right)~ + ~\Delta x~M_{{ac}} ~H_{d} ~} \right]}}$$

Where: Q_r_ is refrigeration output, Q_1−2_ and Q_2−3_ are heat supplied to the generator and c_ac_ and c_r_ are the heat capacities for activated carbon and refrigerant, M_ac_, M_r1_ and M_r, av_ are the masses of activated carbon, refrigerant, and average refrigerant masses respectively, T_1_, T_2_ and T_3_ are the temperatures of generator at start, end of cooling process, and start point of adsorption process, respectively, L_o_ is the latent heat of vaporization of ethanol, ∆M_r_ is actual quantity of uptake, T_3' _and T_4' _are the condensing and evaporating refrigerant temperatures, respectively, and ∆x is the concentration variation.

H_d_ is the average of heat during cooling/heating processes for pair, as presented by Zhao et al.^38^ and Zhao^39^ as in Eq. (2):

2 $$H_{d} = \frac{{~H_{d1} + \,H_{d2} }}{2} = ~~\frac{{\left( {R_{0} *\frac{{T_{1} T_{2} }}{{T_{1} - T_{2} }}*\ln \frac{{P_{c} }}{{P_{e} }}} \right) + \left( {R_{0} *\frac{{T_{3} T_{4} }}{{T_{3} - T_{4} }}*\ln \frac{{P_{c} }}{{P_{e} }}} \right)}}{2}$$

Where: H_d1_, H_d2_ are the heat during cooling and heating processes, T_4_ is the temperature of the generator at end of cooling process, R_o_ is specific gas constant of ethanol, and P_c_, P_e_ are the condenser and evaporator pressure.

Equation (3) divides the cooling output per unit mass of adsorbent to determine the specific cooling effect, which measures the system performance.

3 $$\:\text{S}\text{C}\text{E}=\frac{{\text{Q}}_{\text{r}}}{{\text{M}}_{\text{a}\text{c}}}$$

According to Eq. (4), specific power consumption is the amount of power used or supplied to the generator per unit mass of adsorbent.

4 $$\:\text{S}\text{P}\text{C}=\frac{({\text{Q}\:}_{1-2}+\:{\text{Q}}_{2-3})}{{\text{M}}_{\text{a}\text{c}}}$$

As shown in Eqs. 5, 6 and 7, the exergetic efficiency (thermodynamic efficiency) for the adsorption refrigeration system represented the difference between the actual performance in the present study and the maximum performance as presented by Pons et al.^40^.

5 $$\:\text{E}\text{x}\text{e}\text{r}\text{g}\text{y}\:\text{e}\text{f}\text{f}\text{i}\text{c}\text{i}\text{e}\text{n}\text{c}\text{y}\:=\frac{{\text{C}\text{O}\text{P}\:}_{\text{c}\text{y}\text{c}\text{l}\text{e}}}{{\text{C}\text{O}\text{P}\:}_{\text{m}\text{a}\text{x}}}$$

Where:

6 $$\:{\text{C}\text{O}\text{P}\:}_{\text{c}\text{y}\text{c}\text{l}\text{e}}\:=\frac{{\text{Q}}_{\text{r}}}{{\text{Q}}_{\text{g}\text{e}\text{n}}}$$

7 $$\:{\text{C}\text{O}\text{P}\:}_{\text{m}\text{a}\text{x}}\:=\frac{1-({\text{T}\:}_{\text{a}\text{d}\text{s}\text{o}\text{r}\text{b}\text{e}\text{r}}/{\text{T}\:}_{\text{g}\text{e}\text{n}})\:}{({\text{T}\:}_{\text{a}\text{d}\text{s}\text{o}\text{r}\text{b}\text{e}\text{r}}/{\text{T}}_{\:\text{e}\text{v}})-1}$$

### Uncertainty analysis

The uncertainties related to the measured and computed parameters were affected by a variety of factors, such as the environment, test planning, observation, instrument selection, conditions, and calibration, as illustrated in Table 4. Coleman and Steele^41^ introduced a method for estimating uncertainty that employs the root sum square technique to aggregate individual inputs, as shown in Eq. (8).

For example, the uncertainty of the Coefficient of Performance (COP) of a cycle utilizing an AC/Ethanol through the following steps:

- The values for Q_r_, Q_1−2_, and Q_2−3_ were recorded as 13.08 kJ, 17.12 kJ, and 20.42 kJ, which can be calculated using Eq. (1). The thermo-physical parameters for AC and ethanol are showed in Table 1, while the experimental parameters for the AC/Ethanol are presented in Table 5.
- Subsequently, the uncertainties for Q_r_, Q_1−2_, and Q_2−3_ were determined of values ± 0.147, ± 0.248, and ± 0.261 kJ (1%, 1.45%, and 0.0128%, respectively). These values can be derived by differentiating each parameter according to its respective equations and substituting the uncertainty values of the parameters listed in Table 4.
- Ultimately, the COP value can be calculated using Eq. (6). The uncertainty associated with the COP is ± 0.04 (± 4%) when substituted into Eq. (8).

8 $$U_{{COP}} = \pm \sqrt {{\left( {\frac{{\partial COP}}{{\partial Q_{r} }}U_{{Qr}} } \right)^{2} } + \left( {\frac{{\partial COP}}{{\partial Q_{{12}} }}U_{{Q12}} } \right)^{2} + \left( {\frac{{\partial COP}}{{\partial Q_{{23}} }}U_{{Q23}} } \right)^{2} }$$

**Table 4:** Uncertainty for different operating parameters.

Parameter	Uncertainty (U)
The concentration variation and the latent heat of vaporization of ethanol, and the average of heat (∆x, L_o,_ and H_d_)	± 0.1% (s.d.)
Specific heats of ethanol and AC, (c_r_, and c_ac_)	± 0.1% (s.d.)
Mass of AC, (M_ac_)	± 0.1% (s.d.)
Mass of refrigerant adsorbed, mass of refrigerant, and average refrigerant masses, (∆M_r_, M_r1_ and M_r, av_)	± 1.0% (s.e.m.)
Temperature difference, (∆T)	± 0.5 °C (s.d.)
Heat supplied to the system, (Q_1−2_, and Q_2−3_)	± 1.5% (s.e.m.)
Heat adsorbed in evaporator, (Q_r_)	± 1.0% (s.e.m.)
COP of cycle	± 4.0% (s.e.m.)
Exergy efficiency	± 3.5% (s.e.m.)

### Sample of COP, SCE, and SPC calculations

The calculations for the Coefficient of Performance (COP), Specific Cooling Effect (SCE), and Specific Power Consumption (SPC) of the adsorption refrigeration system were conducted using the equations presented above. The parameter values listed in Table 5 were derived from the experiments carried out in this study. These values pertain to the utilizing the AC/Ethanol pair and the AC with four inclined fins in one sector/Ethanol pair, employing the optimal fin configuration as elaborated in the subsequent section.

To begin with, the duration of the adsorption refrigeration cycle can be established by initiating the process at point 1, denoted as (T_1_), which corresponds to the minimum generator temperature. The process then progresses to point 2 at (T_2_), which is defined upon reaching the condensing pressure indicated by the pressure gauge. Subsequently, point 3 at (T_3_) is attained by continuing the heat supply until the maximum temperature is reached. Finally, point 4 at (T_4_) can be easily identified after achieving the evaporating pressure through cooling. Additionally, T_3'_ and T_4'_ can be ascertained by measuring the ethanol temperature at the condensing and evaporating pressures.

The second step involved the specific values of the average mass of ethanol (M_r av_), the mass of the liquid refrigerant (∆M_r_), and the variation in concentration (∆x). These parameters are established by identifying the minimum ethanol uptake (M_r_ at points 3 and 4) and the maximum ethanol uptake (M_r_ at points 1 and 2), which are obtained from experiments utilizing the integrated liquid indicator. The third step entails calculating the average heat during the cooling and heating processes (H_d_) after determining the values of H_d1_ and H_d2_ through Eq. (2). The final step consists of calculating the quantity of heat that is either supplied or removed during the adsorption and desorption processes. Subsequently, the coefficients of performance (COP), specific cooling effect (SCE), and specific power consumption (SPC) were computed using Eqs. (3, 4, and 6).

**Table 5:** Sample of COP, SCE, and SPC calculations.

Parameter	AC/Ethanol pair	AC - four Inclined fins of one sector/Ethanol pair
T_3'_, ^o^C	22	22
T_4'_, ^o^C	12	6
T_1_, ^o^C	30	31.5
T_2_, ^o^C	65	68
T_3_, ^o^C	82	82
T_4_, ^o^C	60	57
M_r_)_1,2_, kg	0.016	0.025
M_r_)_3,4_, kg	0	0.0016
M_r_)_av_, kg	0.008	0.013
∆x, kg/kg	0.032	0.05
H_d1_, kJ	582	515
H_d2_, kJ	972.6	848.2
H_d_, kJ	777.3	681.6
∆M_r_, kg	0.016	0.023
Q_1−2_, kJ	17.12	18.7
Q_2−3_, kJ	20.4	23.7
Q_3−4_, kJ	4	11.3
Q_r_, kJ	13.1	18.5
SCE, kJ/kg	**26**	**37**
SPC, kJ/kg	**75**	**84.8**
COP	**0.35**	**0.44**

## Results and discussion

### Validation of experiments

**Fig. 9:**
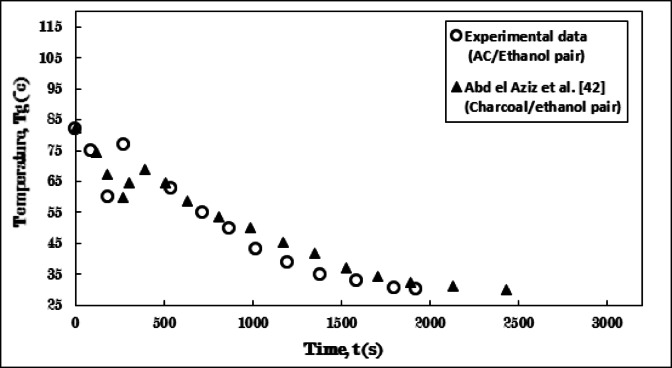
Verification of temperature development over time for the experimental results with data of Abdel Aziz et al.^42^ during the adsorption process.

**Fig. 10:**
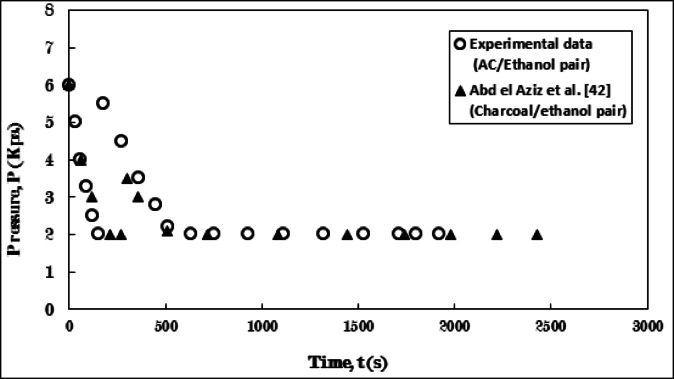
Verification of pressure development over time for the experimental results with data of Abdel Aziz et al.^42^ during the adsorption process.

The measurements of the present study were validated by the results of the AC/Ethanol pair with those reported by Abdel Aziz et al.^42^, who utilized the Charcoal/Ethanol pair. Figure 9 illustrates the temperature progression of the adsorbent within the generator. Additionally, Fig. 10 depicts the pressure evolution between the present study and the previous research.

The trends observed in both figures affirm the validity of the results from the present study in relation to the data provided by Abdel Aziz et al.^42^. The findings from both studies exhibit a high degree of similarity over time, with the exception of certain values that appear to diverge slightly. This can be attributed to the differences in thermo-physical properties, the AC’s favorable response to temperature, which resulted in a 1.5 min variation in the duration of the isosteric cooling process. Furthermore, the intensity of the abrupt temperature increase at the onset of the adsorption process varied, which is associated with a strong compatibility and interaction between the adsorbate and the solid adsorbent for ethanol adsorption. Consequently, an increase in the quantity of adsorbed ethanol leads to a greater release of heat during adsorption and more pronounced temperature fluctuations. Ultimately, the variation in the duration of the adsorption process between the two studies indicates the adsorbent’s capacity to adsorb larger quantities of adsorbate, significantly influenced by its specific surface area and total pore volume for each solid adsorbent.

### Experimental observations

The adsorption refrigeration system utilizes activated carbon and ethanol as its working pair. The primary objective of the experimental study was to enhance the system’s performance by increasing the heat and mass transfer within the generator. Four aluminum fins are placed into the adsorbent column in a radial orientation to ensure contact with the inner surface of the generator. The experiments were carried out at generating and evaporating temperatures of 82 °C and 5 °C, respectively.

The parameters measured during the experiments for the cooling-adsorption process are detailed in Table 6. Initially, the evaporator was set to a condensing pressure of 6 kPa, corresponding to a temperature of 22 °C. The lowest evaporating temperature of the adsorption refrigeration system was recorded using fins of 3 sectors configuration, achieving a decreasing rate of 19.5 °C.

In the second configuration, inclined fins in one sector resulted in a reduction in the evaporating temperature of 16 °C, followed by straight fins in one sector, fins in 2 sectors, fins in 4 sectors, the pure AC case, and finally, fins in 6 sectors yielding values of 15.25 °C, 13.4 °C, 13 °C, 10 °C, and 9 °C, respectively, as illustrated in Table 6. These results suggest that the optimal configuration for fins is in 3 sectors, which achieves the highest reduction in evaporating temperature. The improvement in the reduction is approximately a 9.5 °C difference when compared to the pure AC without fins.

The duration of the cooling-adsorption cycle is a crucial element that greatly influences the performance of the system. The minimum duration is recorded at 22 min for both straight and inclined fins in one-sector configurations, whereas the fins in the 3-sector configuration take 33 min. This increase in duration for the 3-sector fins is attributed to the improved heat transfer through the generator and among the adsorbent layers, which enhances the mass transfer of refrigerant within the generator, leading to a reduction in the evaporating temperature. Consequently, this configuration requires additional time to reach a complete halt and achieve equilibrium at the maximum uptake capacity. Furthermore, the inclined fins configuration demonstrates a slight advantage over the straight fins in a one-sector setup, with a reduction rate of 0.75 °C in the evaporating temperature. This indicates superior heat distribution among the adsorbent layers, resulting in a marginal improvement in both heat and mass transfer throughout the cycle.

Thus, the maximum uptake capacity is with the 3-sector fins at 0.06 kg/kg, followed by inclined fins, straight fins, fins of 2 sectors, fins of 4 sectors, pure AC without fins, and finally, the 6-sector fins with values of 0.05 kg/kg, 0.045 kg/kg, 0.043 kg/kg, 0.04 kg/kg, 0.032 kg/kg, and 0.024 kg/kg, respectively, as illustrated in Table 6. The 6-sector fins configuration exhibits the lowest uptake capacity alongside a prolonged cycle time. This is ineffective for achieving satisfactory performance when juxtaposed with the finless AC.

**Table 6:** Experimental measured parameters during the adsorption process of AC without fins and with fins in six different configurations.

Case No.	Generator with six configurations of fins and without fins	Reduction in evaporator temperature, ΔT_e_, (˚C)	Cooling-adsorption cycle time, (min.)	Uptake amount, ΔX, (kg/kg)
–	Activated carbon (without fins)	10	32	0.032
1	Fins of one sector, (Straight fins)	15.25	22	0.045
2	Fins of one sector, (Inclined fins)	16	22	0.05
3	Fins of two sectors	13.4	27.5	0.043
4	Fins of three sectors	19.5	33	0.06
5	Fins of four sectors	13	27	0.04
6	Fins of six sectors	9	30	0.024

As a result, the performance of the adsorption refrigeration system are influenced by the aforementioned findings. The highest heat adsorbed was recorded with fins of three sectors configuration, achieving 22.25 kJ, followed by inclined fins in one sector, straight fins in one sector, fins in two sectors, fins in four sectors, the finless AC, and fins in six sectors have values of heat adsorbed (Q_r_) equal 18.5 kJ, 17.7 kJ, 17 kJ, 16.2 kJ, 13 kJ, and 9.8 kJ, respectively. The quantity of heat adsorbed (Q_r_) serves as a measure of the extent of ethanol adsorption (ΔX), thus ensuring that the six different configurations retain the same sequence. Consequently, a significant level of adsorption correlates with a substantial quantity of heat adsorbed.

The increase in the heat supplied to the system during the heating-desorption process adversely impacts the system’s performance and efficiency. In the isosteric heating process (1–2), the supplied heat values were 17.12 kJ, 18.3 kJ, 18.7 kJ, 19.7 kJ, 22.8 kJ, 17.5 kJ, and 16.8 kJ for the finless AC, straight fins in one sector, inclined fins in one sector, fins in two sectors, fins in three sectors, fins in four sectors, and fins in six sectors, respectively. Furthermore, the heat supplied during the desorption process (2–3) for the above arrangement of fins configurations and finless AC were 20.4 kJ, 23 kJ, 23.7 kJ, 26.7 kJ, 28.9 kJ, 23.7 kJ, and 17.4 kJ, respectively.

As illustrated in Table 7, the maximum heat supplied was to fins of three sectors configuration, totaling 51.7 kJ, followed by fins of two sectors, inclined fins of one sector, straight fins of one sector, fins of four sectors, the finless AC, and fins of six sectors, with values of 46.4 kJ, 42.4 kJ, 41.3 kJ, 41.2 kJ, 37.5 kJ, and 34.2 kJ, respectively. The configuration of fins in three sectors consumed the highest amount of heat, facilitating ethanol for recapture in the evaporator during desorption process. Therefore, a greater adsorbed amount of ethanol correlates with an increased in supplied heat to the system. In relation to the heat supplied, the arrangement of the fins configurations presented above have the same order of their uptake amount.

Achieving balance entails minimizing the disparity between the heat values supplied and adsorbed, which led to the inclined fins of one sector attaining the highest coefficient of performance (COP) and optimal system efficiency. The configuration of inclined fins of one sector recorded the highest COP at a value of 0.44, followed by straight fins of one sector and fins of three sectors, which exhibited identical values, along with fins of four sectors, fins of two sectors, finless AC, and finally, fins of six sectors, which had values of 0.43, 0.39, 0.37, 0.35, and 0.29, respectively, as illustrated in Table 7.

Regarding the arrangement of COP, variations were observed in the previously discussed order, which were associated with the quantity of refrigerant uptake. The equality in COP values for straight fins of one sector and fins of three sectors provides them with an equivalent option when prioritizing high system performance. However, when considering the refrigeration effect at a lower evaporating temperature, fins of three sectors are preferred over straight fins of one sector.

The enhancement in COP and efficiency of the adsorption refrigeration system utilizing finned (inclined fins of one sector) adsorber achieved rates of 25.7% and 25%, respectively, in comparison to finless adsorber.

**Table 7:** Experimental calculated performance parameters of the adsorption refrigeration system for AC without fins and with fins in six different configurations.

Case No.	Generator with six configurations of fins and without fins	Heat adsorbed, Q_*r*_, (kJ)	Heat supplied, Q_in_, (kJ)	COP	Efficiency %
–	Activated carbon (without fins)	13	37.5	0.35	20.3
1	Fins of one sector, (straight fins)	17.7	41.3	0.43	24.9
2	Fins of one sector, (inclined fins)	18.5	42.4	0.44	25.4
3	Fins of two sectors	17	46.4	0.37	21.4
4	Fins of three sectors	22.25	51.7	0.43	24.9
5	Fins of four sectors	16.2	41.2	0.39	22.5
6	Fins of six sectors	9.8	34.2	0.29	16.8

The existence of fins typically shortens the length of the adsorption cycle, and the quantity of adsorption is a crucial factor that significantly influences the system’s efficiency and is directly associated with the reduction in evaporator temperature, as demonstrated in the analyzed cases.

### Variation of generator, evaporator temperatures, and uptake amount for the experiments studied

In conditions that are transient and characterized by non-uniform pressure, the adsorption-desorption refrigeration cycle was implemented, and the experiments were carried out. The dynamic flow of the refrigerant is influenced by the driving forces of adsorption and desorption that exist between the granules of the adsorbent and the adsorbate as a working pair, which aids in this process. The temperatures of the generator and evaporator, along with the quantity of ethanol adsorbed, are recorded using T-type thermocouples and a graduated liquid indicator to assess the performance of the adsorber with pure AC (without fins) and six distinct fin configurations.

**Fig. 11:**
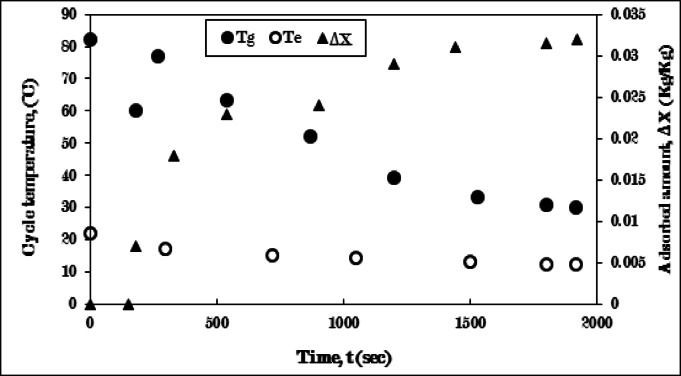
Variation of generator (T_g_), evaporator (T_e_) temperatures, and adsorbed amount (ΔX) with time for Pure AC (without fins)/Ethanol pair during cooling-adsorption process at Tg = 82 °C, and Te = 5 °C.

Figure 11 depicts the cooling-adsorption process employing a pure AC/Ethanol pair, without fins in the generator. Throughout the cooling phase (3–4), the generator’s temperature sharply drops from 82 °C to 60 °C, while the evaporator’s temperature sees a minor decrease, with no refrigerant adsorption occurring, for a period of 180 s. The reduction in the generator’s temperature is due to the cooling effect provided by the cold water circulating within the water jacket surrounding the generator. Although the evaporator’s temperature remains relatively stable and there is no ethanol adsorption during this process, this is because the valve connecting the evaporator and generator remains closed throughout the cooling process (3–4). Subsequently, the generator’s temperature undergoes a notable rise, followed by a notable decrease until 1500 s, then decreases gradually until it reaches a stable state at 30 °C after 1920 s.

The temperature trajectory of the generator is attributed to the initiation of the adsorption process (4 − 1). This process commences with the opening of the valve that links the evaporator to the generator, resulting in a temperature increase. This rise is a consequence of the pressure differential between the two components, along with the heat of adsorption that released during the adsorption process upon initial contact. The ongoing reduction in the generating temperature is associated with the cooling effect produced by the cold water circulating within the generator’s water jacket. Simultaneously, the temperature of the evaporator consistently declines from 22 °C to 10 °C. This decline is linked to the rising quantity of adsorbed ethanol, which leads to a decrease in the evaporator temperature, continuing until it stabilizes at the conclusion of the adsorption process, characterized by the lowest evaporating temperature and the maximum uptake amount.

The uptake amount experiences a sharp increase, followed by a gradual rise until it attains a peak value of 0.032 kg/kg after 1920 s, as illustrated in Fig. 11. The limited quantity of the uptake amount referred to the poor thermal conductivity of the finless adsorber, so, the existence of fins is required to improve the thermal conductivity of the adsorber as the discussion in the following results.

**Fig. 12:**
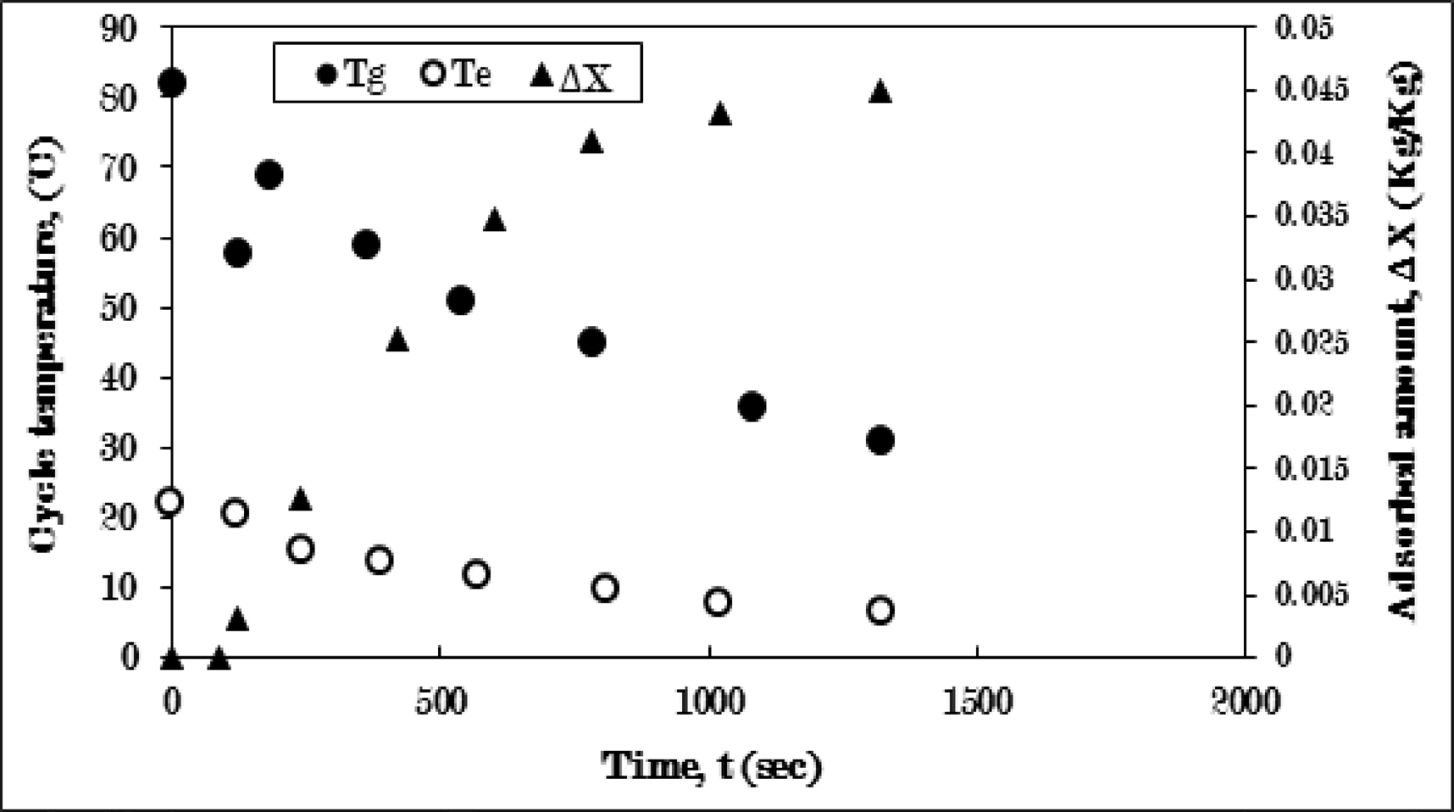
Variation of generator (T_g_), evaporator (T_e_) temperatures, and adsorbed amount (ΔX) with time for AC-straight fins of one sector/Ethanol pair during cooling-adsorption process at T_g_ = 82 °C and T_e_ = 5 °C.

Figure 12 depicts the developments in generator and evaporator temperatures, as well as the quantity of adsorbed ethanol, utilizing AC-straight fins of one sector/Ethanol combination throughout the cooling-adsorption process (3-4-1). The cooling process witnesses a decline from the generator’s starting temperature of 82 °C to 58 °C for 120 s. Additionally, the adsorption process concludes in 1320 s. It is evident that the cooling process duration is shortened by one minute, and the total process time is reduced by approximately 10 min when compared to the experiment utilizing pure AC (without fins). This decrease in process time is attributed to the presence of aluminum straight fins that were radially inserted within the AC granule, enhancing the thermal conductivity between the generator’s inner surface and the adsorbent granules.

The evaporator temperature progressively diminishes from 22 °C to 6.75 °C, resulting in a difference of 5.25 °C lower than the pure AC scenario. The more significant reduction in evaporating temperature correlates with a higher quantity of ethanol adsorbed during the adsorption phase, amounting to 0.045 kg/kg.

This phenomenon can be explained by the enhancement of heat and mass transfer among the adsorbent layers, achieved by minimizing the contact surface resistance between the bed surface and the solid adsorbent.

The effect of using the straight fins in different orientation angle (45˚ from the radial orientation) as discussed in the following experimental results.

**Fig. 13:**
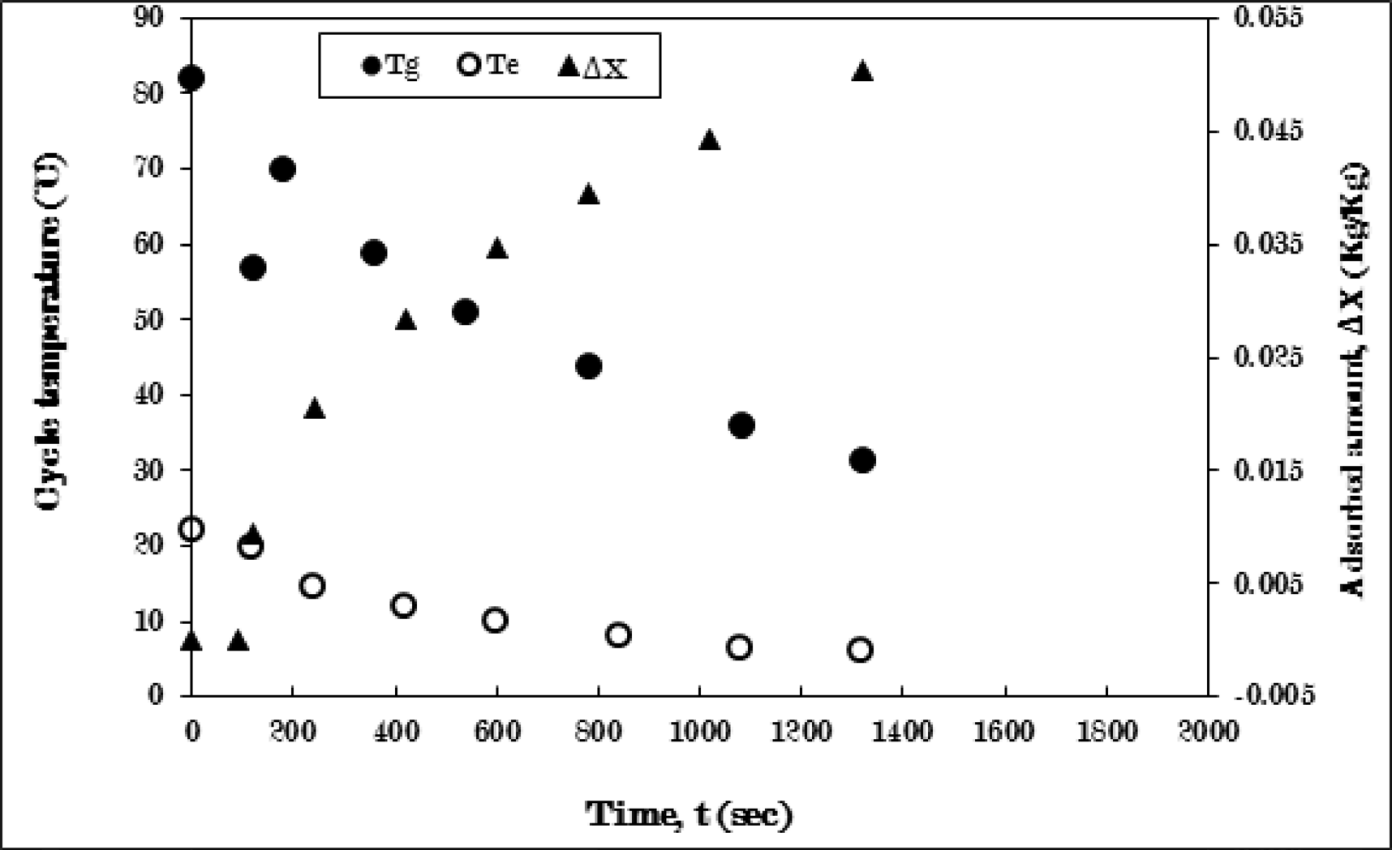
Variation of generator (T_g_), evaporator (T_e_) temperatures, and adsorbed amount (ΔX) with time for AC-Inclined fins of one sector **/**Ethanol pair during cooling-adsorption process at T_g_ = 82 °C and T_e_ = 5 °C.

Figure 13 illustrates the temperatures of the generator and evaporator, as well as the amount of adsorbed ethanol during the cooling-adsorption process involving the AC-inclined fins/Ethanol combination. The aluminum fins, positioned at a 45-degree angle, are integrated within the AC to ensure optimal contact with the generator’s inner surface.

During the cooling phase, the temperature of the generator drops from 82 °C to 57 °C over 120 s, while the adsorption process concludes after 1320 min. The evaporating temperature experiences a reduction of approximately 16 °C throughout the adsorption process compared with pure AC. Additionally, the quantity of ethanol absorbed rises to 0.05 kg/kg. A minor enhancement in the configuration of the inclined fins results in a decrease in the evaporating temperature by 0.75 °C and an uptick in the adsorption amount by 0.005 kg/kg when compared to straight fins configuration. Despite both fin designs exhibiting similar properties, the difference in angle produces distinct results. This improvement can be attributed to the increased heat and mass transfer within the adsorbent, facilitated by the fins’ inclined orientation, which enhances heat diffusivity compared to the radial direction achieved by straight fins.

Consequently, the inclined fins demonstrate higher effectiveness in enhancing the performance of the adsorption system relative to the straight fins.

**Fig. 14:**
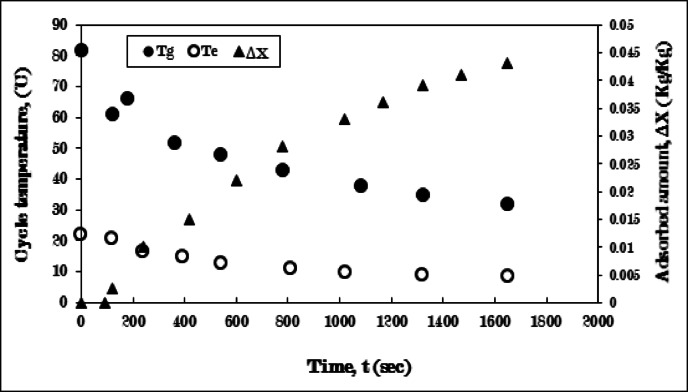
Variation of generator (T_g_), evaporator (T_e_) temperatures, and adsorbed amount (ΔX) with time for AC-fins of two sectors **/**Ethanol pair during cooling-adsorption process at T_g_ = 82 °C and T_e_ = 5 °C.

The AC-fins of two sectors/Ethanol pair were utilized in the adsorption refrigeration system. The temperatures of the generator and evaporator, along with the uptake amount during the cooling-adsorption process, are illustrated in Fig. 14. The temperature of the generator decreases from 82 °C to 61 °C within 120 s, continuing to decline to 32 ˚C over 1650 s during the adsorption process. The reduction in the evaporating temperature to approximately 13.4 °C. Furthermore, the uptake amount is around 0.043 kg/kg, which corresponds to the pure AC uptake value.

The extension of the cycle duration and the decrease in the reduction of the evaporating temperature, in conjunction with the diminished uptake amount relative to the fins of a single sector, whether straight or inclined, can be ascribed to the ineffective influence exerted by the presence of fins of two sectors within the generator. Suboptimal values occur as a result of inadequate heat and mass transfer within the adsorbent layers, stemming from the relatively restricted thermal diffusion between these layers. Wide-angle two-sector fins are unable to surpass the equivalent thermal conduction resistance present between solid grains when compared to the efficacy of single-sector fins. This suggests that the heat transferred radially through the fins is less than the heat diffusion occurring within the generator, which results in an extended adsorption cycle time for this configuration.

This configuration resulted in a modest enhancement in cycle performance relative to the pure AC. Additionally, a markedly greater cooling effect was noted in this configuration in comparison to the pure AC case; however, it was still inferior to that of the one sector fin configuration, regardless of whether it was straight or inclined.

These results renders this configuration of minimal significance. Consequently, the subsequent experiment investigates the impact of augmenting the fin cross-sections across three sectors on the cycle performance.

**Fig. 15:**
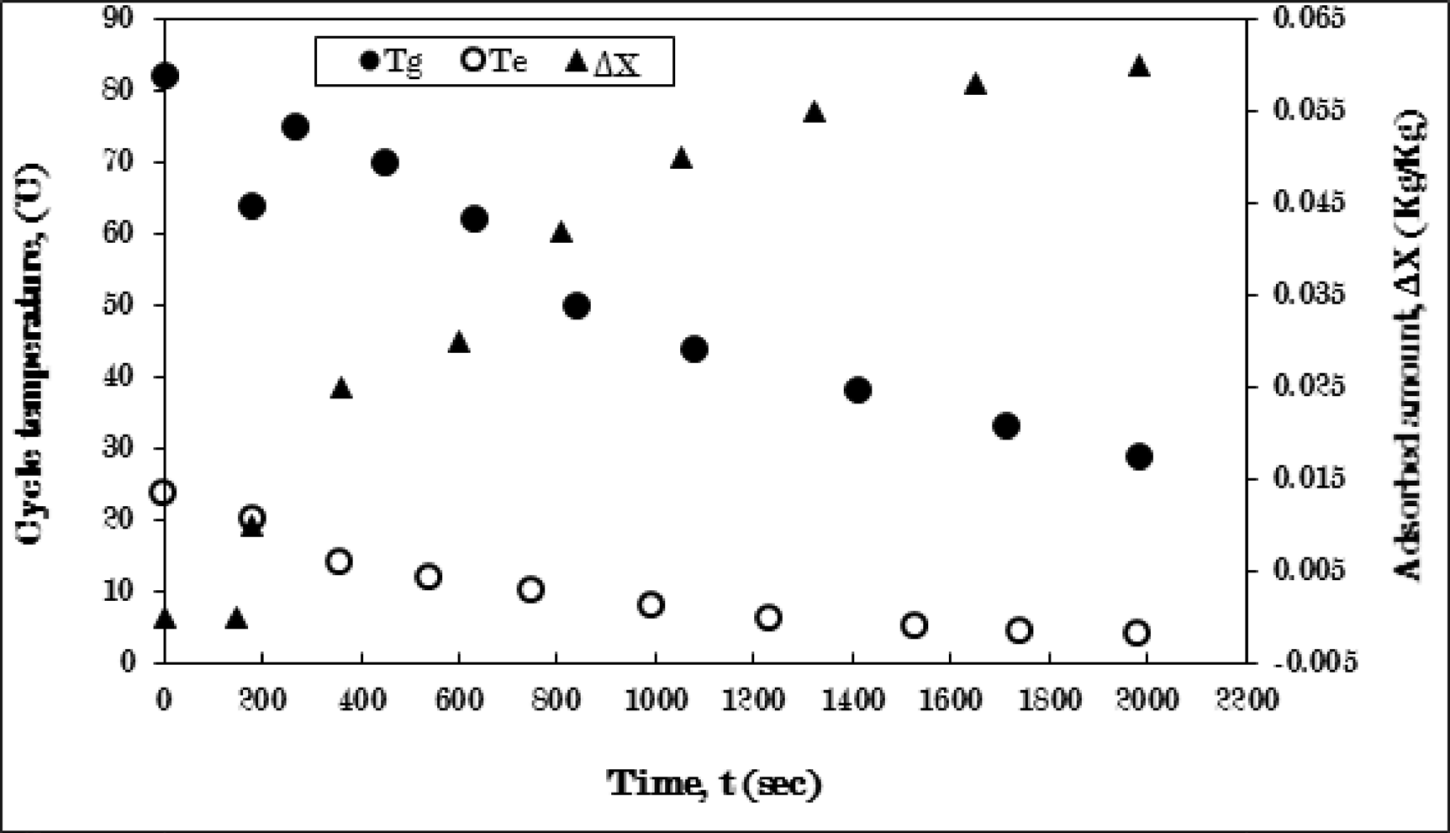
Variation of generator (T_g_), evaporator (T_e_) temperatures, and adsorbed amount (ΔX) with time for AC-fins of three sectors **/**Ethanol pair during cooling-adsorption process at T_g_ = 82 °C and T_e_ = 5 °C.

In contrast, the fins configured in a three-sector arrangement exhibit a significant improvement in cycle performance, as indicated by a notable rise in the generator’s temperature at the beginning of the adsorption process related to a considerable amount of ethanol adsorbed, and the adsorbed amount continues to increasing until the adsorption process stops. The cooling process lasted 180 s until it reached 64 °C, while the adsorption process was completed in 1980 s. Furthermore, the temperature of the evaporator saw a marked decrease, falling below 5 °C until the process was halted, as illustrated in Fig. 15. The amount of uptake follows a similar enhancement, achieving 0.06 kg/kg, which is nearly double the uptake value recorded in the pure AC case. The increase in uptake amount was 87.5% compared with pure AC case.

The use of three-sector fins greatly improves heat and mass transfer within the generator, the limited values of the temperature gradient within the generator exist due to the heat of adsorption released when the ethanol migrate between the adsorbent layers during the adsorption process, that results in a more significant evaporating temperature drop during the adsorption process. This enhancement leads to a greater amount of adsorbed ethanol, which extends the time necessary for the granules to reach full saturation with ethanol, thereby lengthening the adsorption process duration.

It is crucial to uphold quantity of aluminum fins utilized in the generator to prevent adverse effects on the circuit’s performance by increase the aluminum quantity. This determination is made according to the specifications of the fins (length, width, thickness, quantity, and shape), taking into account the influence of each factor on the performance of the cycle prior to the decision to implement a finned adsorber. In the three-sector fin configuration, this arrangement matches the required heat diffusion rate within the solid adsorber, which exhibits a temperature gradient in the angular direction within the adsorbent layers, enhancing mass diffusion within the generator and improving cycle performance.

The following experiment entails raising the number of fin sectors to four, with the objective of improving heat transfer within the generator.

**Fig. 16:**
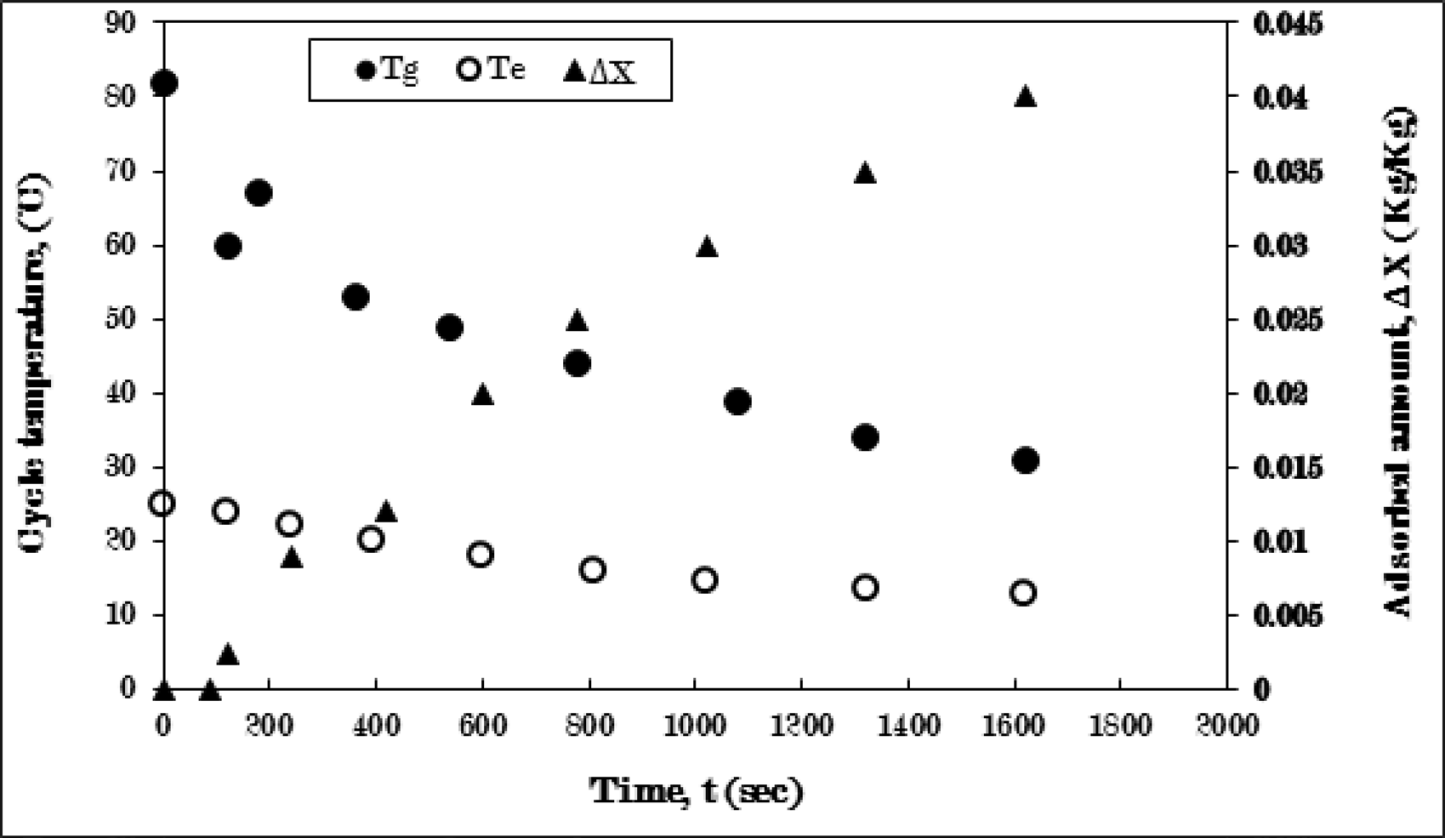
Variation of generator (T_g_), evaporator (T_e_) temperatures, and adsorbed amount (ΔX) with time for AC-fins of four sectors **/**Ethanol pair during cooling- adsorption process at T_g_ = 82 °C and T_e_ = 5 °C.

In Fig. 16, the temperatures of the generator and evaporator, along with the amount of uptake, are illustrated using fins of four sectors configuration. The cooling process lasts for 120 s at a temperature of 62 °C. Furthermore, the adsorption process is finalized and halted at 1620 s and 31 °C. The rate of decrease in the evaporating temperature is recorded of 13 °C, while the peak uptake amount of ethanol reaches 0.04 kg/kg.

These outcomes can be attributed to the presence of fins of four sectors that were incorporated within the generator. There is to the heat transfer through the fins more than the rate of heat diffusivity within the generator, albeit with a reduction in mass transfer. Consequently, resulting in a relatively modest uptake amount. This configuration yields outcomes that are close to those obtained with two-sector fins, with a notable enhancement in the system efficiency.

The concluding experiment will utilize six sector fins. No enhancement was noted with the increase in fin sections as presented in the earlier experiment. So, a reduction in cycle performance is anticipated with six sector fins. This highlights the significance of balancing the quantity of metal with the area needed for improvement.

**Fig. 17:**
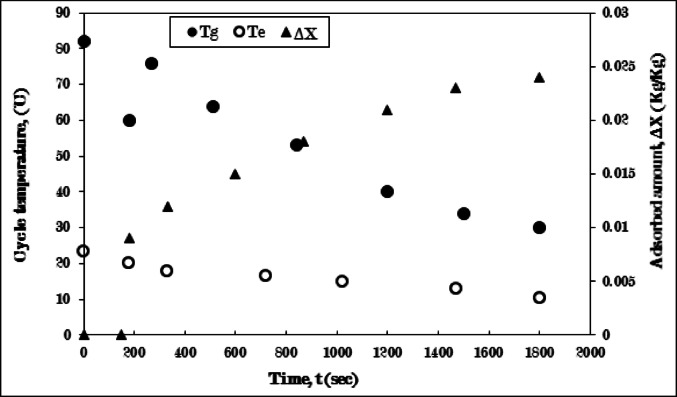
Variation of generator (T_g_), evaporator (T_e_) temperatures, and adsorbed amount (ΔX) with time for AC-fins of six sectors **/**Ethanol pair during cooling-adsorption process at T_g_ = 82 °C and T_e_ = 5 °C.

In Fig. 17, the effect of utilizing fins in a six-sector configuration is illustrated. The temperatures of the generator and evaporator, along with the quantity of ethanol uptake, are displayed. The cooling process lasted for 180 s at a temperature of 60 °C, while the adsorption process concluded at 1800 s. The evaporating temperature experienced a reduction of 9 ˚C, with the ethanol uptake amount to 0.024 kg/kg.

The extended duration of both the cooling and adsorption processes is attributed to the influence of the six-sector fins, which markedly enhance heat transfer across the fins, surpassing the heat diffusion within the solid adsorbent. Consequently, this leads to a diminished thermal gradient within the layer, where the temperature of the adsorbent granules adjacent to the fins is lower than that of the granules located at the midpoint between any two fins. Furthermore, the mass transfer occurring within the generator diminishes, resulting in a minimal quantity of ethanol being adsorbed within the generator, which leads to a slight reduction in the evaporating temperature.

This fin configuration exemplifies the least appropriate arrangement for a generator. This is attributed to the elevated quantity of aluminum present within a limited space in the generator, which leads to inadequate heat and mass transfer inside the generator, as discussed in the previous paragraph. The outcomes of this configuration are inferior to those seen with pure AC regarding the adsorbed quantity and performance coefficient. Consequently, it is deemed unsuitable for application in generators.

Among the configurations analyzed, the inclined one-sector fins exhibited better performance regarding the cycle coefficient of performance, whereas the three-sector fins were outstanding in attaining the maximum cycle refrigeration effect.

### COP and SCE of adsorption refrigeration system for experiments studied

**Fig. 18:**
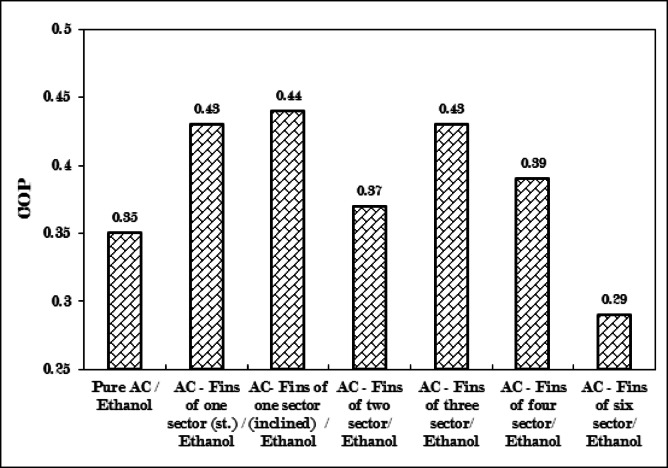
Comparison between coefficient of performance (COP) for Pure AC and AC with six different configurations of fins.

**Fig. 19:**
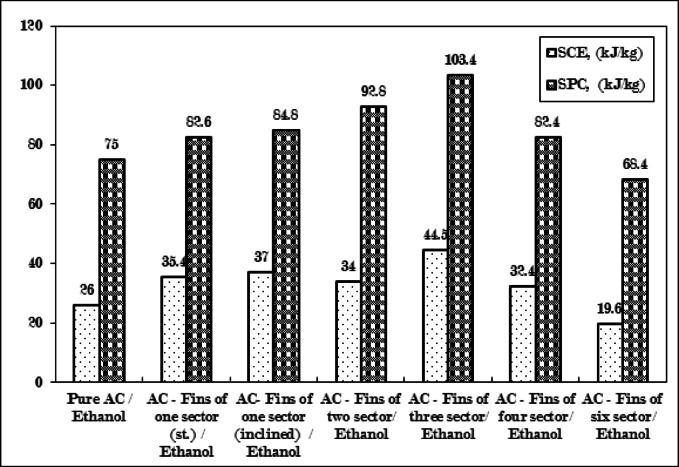
Comparison between specific cooling effect (SCE) and specific power consumption (SPC) for pure AC and AC with six different configurations of fins.

The coefficient of performance, specific cooling effect, and specific power consumption are elaborated upon in Figs. 18 and 19. The inclined fins of one sector produced the highest coefficient of performance (COP) at 0.44, accompanied by a specific cooling effect (SCE) of 37 kJ/kg and a specific power consumption (SPC) of 84.8 kJ/kg. Closely following, the straight fins of one sector - the three-sector fins attained a COP of 0.43. Subsequently, the fins of four sectors, the fins of two sectors, pure AC, and the fins of six sectors recorded values of 0.39, 0.37, 0.35, and 0.29, respectively.

The maximum COP value attained by the inclined fins of one sector results from achieving an optimal balance between the quantity of heat adsorbed and the quantity of heat supplied to the adsorption refrigeration cycle, thereby minimizing the difference between these two values.

Furthermore, the constrained SPC value of 84.8 kJ/kg plays a significant role in improving the system’s coefficient of performance. All these factors are influenced by the brief cycle time, substantial uptake amount, and the lowest evaporating temperature.

In the configuration involving fins of three sectors, the coefficient of performance (COP) of the adsorption refrigeration system was notably influenced by the specific power consumption, which was approximately 103.4 kJ/kg. Despite the uptake amount being 0.06 kg/kg, it plays a role in improving the specific cooling effect, which reaches 44.5 kJ/kg, consequently leading to an increased cycle duration. Therefore, the optimal cooling effect is achieved with the configuration of fins in three sectors, making it the most advantageous option.

The maximum value of the SCE was attained by fins of three sectors, followed by inclined fins of one sector, straight fins of one sector, fins of two sectors, fins of four sectors, pure AC, and fins of six sectors, yielding values of 44.5 kJ/kg, 37 kJ/kg, 35.4 kJ/kg, 34 kJ/kg, 32.4 kJ/kg, 26 kJ/kg, and 19.6 kJ/kg, respectively.

In contrast, the highest SPC is realized by fins of three sectors, succeeded by fins of two sectors, inclined fins of one sector, straight fins of one sector, fins of four sectors, pure AC, and fins of six sectors, with values of 103.4 kJ/kg, 92.8 kJ/kg, 84.8 kJ/kg, 82.6 kJ/kg, 82.4 kJ/kg, 75 kJ/kg, and 68.4 kJ/kg, respectively. The superior values of these two parameters indicate enhanced system performance compared to lower values, signifying an improvement in the efficiency of the adsorption refrigeration system.

In summary, the six different fin configurations integrated into the adsorption refrigeration system exhibit varying degrees of enhancement. Four configurations (fins of one sector at both straight and inclined angles, as well as fins of three and four sectors) demonstrate an increase in system performance when compared to the pure AC scenario. This enhancement can be attributed to the fins’ capacity to elevate heat and mass diffusivity within the generator, thereby overcoming the resistance present between the adsorbent layers, facilitating a greater migration of ethanol through the system. Consequently, the improvement was observed through a reduction in cycle time alongside a higher adsorbate quantity and an enhanced refrigeration effect.

## Conclusions

In conclusion, an experimental investigation was carried out using an activated carbon (AC)/ethanol pair under transient conditions and non-uniform pressures. The use of valves that controlled the adsorption refrigeration system, significantly reduced heat losses during the adsorption-desorption process, thus improving the precision of the results obtained.

To tackle the issues related to mass diffusion resistance and to enhance thermal diffusivity, a finned generator was employed in the study. The effectiveness of this finned generator was compared to that of a non-finned generator, utilizing six different fin configurations: straight fins in one sector, inclined fins in one sector, fins of two sectors, fins of three sectors, fins of four sectors, and fins of six sectors.

The configurations inclined fins in one sector, straight fins in one sector, fins of three sectors demonstrate superior performance, followed by fins of four sectors and two sectors when compared to pure AC, and finally, fins with six sectors, yielding values of 0.44, 0.43, 0.43, 0.39, 0.37, 0.35, and 0.29, respectively. The specific values of SCE and SPC for the three sector fins, inclined fins in one sector, straight fins in one sector, as well as fins with four sectors, two sectors, pure AC, and fins of six sectors are (44.5 and 103.4 kJ/kg), (37 and 848 kJ/kg), (35.4 and 82.6 kJ/kg), (34 and 92.8 kJ/kg), (32.4 and 82.4 kJ/kg), (26 and 75 kJ/kg), and (19.6 and 68.4 kJ/kg), respectively. The efficiency of the cycle decreases when a quantity of metallic fins concentrated in a specific area surpasses the suitable limit, as in the fin configurations (four and six sectors), the heat transfer rate increases than the mass transfer rate within the spaces of fins. This explains the reason of their less performance.

The examination of three-sector fins featuring a specific cooling effect (SCE) of 44.5 kJ/kg and an adsorbed quantity of 0.06 kg/kg revealed a significant improvement in both the specific cooling effect and the uptake amount with reduced evaporating temperatures.

The utilization of three-sector fins led to enhancements of 71.2% in SCE and 87.5% in uptake amount, alongside a reduction in evaporating temperature by 95% when compared to the performance of the generator without fins.

Moreover, inclined fins within a single sector enhance the coefficient of performance (COP) to 0.44 for the adsorption refrigeration system. In assessing the system’s efficiency relative to a generator without fins, the introduction of inclined fins of one sector resulted in a 25.7% increase in COP.

## Data Availability

The authors declare that the data supporting the findings of this study are available within the paper, its supplementary information files.

## List of Symbols

Q_r_: Refrigeration output (kJ)
Q_1-2_: Heat supply to generator in process 1-2 (kJ)
Q_2-3_: Heat supply to generator in process 2-3 (kJ)
Q_gen_, Q_in_: Heat supply to generator (kJ)
C_ac_: Specific heat of activated carbon (kJ/kg.K)
C_r_: Specific heat of refrigerant (kJ/kg.K)
M_ac_: Mass of activated carbon (kg)
M_r1_: Mass of refrigerant (kg)
M_r, av_: Average mass of refrigerant (kg)
T_3'_, T_4'_: Condensing and evaporating refrigerant temperatures (^o^C)
L_o_: Latent heat of evaporation (kJ/kg)
∆M_r_: Actual quantity of uptake (kg)
∆x: Concentration ratio variation (kg/kg)
ΔT_e_: Evaporating temperature rate (^o^C)
R_0_: Specific gas constant of ethanol (kJ/kg .K)
H_d_: Average of heat during cooling/heating processes (kJ/kg)
U: Uncertainty

## Subscript

sec: Seconds
min.: Minutes
e: Evaporation
g: Generation
max: Maximum
1, 2, 3, 4: Status of relevant processes
s: Solar

## Abbreviations

ART: Adsorption refrigeration tube
COP: Coefficient of performance
SCE: Specific cooling effect
SPC: Specific power consumption
CFD: Computational fluid dynamic
SS: Stainless steel
AC: Activated carbon
